# Discovery of potential biomarkers in human melanoma cells with different metastatic potential by metabolic and lipidomic profiling

**DOI:** 10.1038/s41598-017-08433-9

**Published:** 2017-08-18

**Authors:** Hye-Youn Kim, Hwanhui Lee, So-Hyun Kim, Hanyong Jin, Jeehyeon Bae, Hyung-Kyoon Choi

**Affiliations:** College of Pharmacy, Chung-Ang University, Seoul, 06974 Republic of Korea

## Abstract

Malignant melanoma, characterized by its ability to metastasize to other organs, is responsible for 90% of skin cancer mortality. To investigate alterations in the cellular metabolome and lipidome related to melanoma metastasis, gas chromatography-mass spectrometry (GC-MS) and direct infusion-mass spectrometry (DI-MS)-based metabolic and lipidomic profiling were performed on extracts of normal human melanocyte (HEMn-LP), low metastatic melanoma (A375, G361), and highly metastatic melanoma (A2058, SK-MEL-28) cell lines. In this study, metabolomic analysis identified aminomalonic acid as a novel potential biomarker to discriminate between different stages of melanoma metastasis. Uptake and release of major metabolites as hallmarks of cancer were also measured between high and low metastatic melanoma cells. Lipid analysis showed a progressive increase in phosphatidylinositol (PI) species with saturated and monounsaturated fatty acyl chains, including 16:0/18:0, 16:0/18:1, 18:0/18:0, and 18:0/18:1, with increasing metastatic potential of melanoma cells, defining these lipids as possible biomarkers. In addition, a partial-least-squares projection to latent structure regression (PLSR) model for the prediction of metastatic properties of melanoma was established, and central metabolic and lipidomic pathways involved in the increased motility and metastatic potential of melanoma cells were identified as therapeutic targets. These results could be used to diagnose and control of melanoma metastasis.

## Introduction

Melanoma is by far the most lethal form of cutaneous cancer because of ability to metastasize to other organs^1^. The global incidence of melanoma, especially among white populations, has been steadily increasing for the past few decades, and 90% of skin cancer mortality is caused by melanoma^2, 3^. Early-stage melanoma, which is located only at the primary site, is curable by surgical resection, and the 5-year survival rate is over 98%. However, if the melanoma has spread to distant sites, including lungs, liver, brain, or lymph nodes, it is refractory to existing therapies, and the 5-year survival rate declines to 17%^4^. Thus, early detection of melanoma and determination of its metastatic potential are important factors in early, more effective treatment. Correspondingly, there is a need for new diagnostic and therapeutic strategies.

Molecular biomarkers can be used to predict carcinogenesis and cancer progression, development of metastases, and therapeutic efficacy. In particular, several biomarkers associated with metastatic dissemination of melanoma have been investigated for their prognostic value and application to the treatment of patients. The level of serum lactate dehydrogenase (LDH), which is translationally upregulated by oncogenes, has been recognized as an important indicator of the metastatic developmental stage of melanoma^5, 6^. Overexpression of human epidermal growth factor receptor 3 (HER3) has been associated with melanoma metastases, and suppression of HER3 inhibits melanoma cell proliferation, migration, and invasion^7^. Additionally, potential biomarkers such as VEGF, MIA, and CXCL1 are known to play a critical role in remodeling of the tumor microenvironment during metastasis of melanoma, and increased levels of these proteins are correlated with more advanced stages of cancer^8–10^.

Metabolomics has emerged as a powerful tool for the discovery of biomarkers in various cancer types such as breast, gastric, lung, and colorectal cancer, because metabolic profiles closely reflect the environmental impact, developmental stage of disease, and response to therapy^11–15^. Previous studies have assessed metabolic change in melanoma using various analytical techniques in *in-vivo* and *in-vitro* models. Comparative metabolite profiling between melanoma and non-melanoma lesions in patients, and profiling of metabolic fluxes of human melanoma cells and melanocytes using gas chromatography-mass spectrometry (GC-MS) have been reported^16, 17^. In addition, metabolic changes related to metastasis of melanoma in a C57BL/6J mouse model and murine melanoma cell lines (Tm1, Tm5, and B16F10) have been identified by nuclear magnetic resonance (NMR)-based metabolic profiling^18–21^, and a fatty acids and phospholipid group analysis of high- and low-metastatic B16 melanoma cell lines by gas-liquid chromatography equipped with dual-flame ionization detectors has been performed^22^. However, there has been no research focused on comparing changes in metabolites and individual lipid species in human melanoma cell lines with low and high metastatic potential with mass spectrometric-based approaches.

In the present study, we hypothesized that matabolic and lipidomic profiling may provide information that discriminate melanoma cells with different metastatic potential and can be used to predict metastatic properties of melanoma for early diagnosis. To test the hypothesis, metabolic and lipidomic profiles of primary melanocytes (HEMn-LP) and melanoma cell lines (A375, A2058, G361, SK-MEL-28) with different metastatic potential were analyzed by GC-MS and direct infusion-mass spectrometry (DI-MS). A375 and A2058 melanoma cells have been previously described as low metastatic and high metastatic cell lines, respectively^23–26^. Lipid changes in metastatic cancer cells are central to metastatic processes such as cell-cell interactions, adhesion to the other organs, and invasion^27–29^. Existing studies on lipids in melanoma cells have only examined the total lipid content and lipid composition in metastatic B16 melanoma cells^22^, whereas the profile of intact lipid species is unknown. Therefore, the objective of this study was to extend the lipidomic analysis to examine changes in intact lipids with various combinations of head groups and fatty acyl chains. In addition, melanoma cell metabolism was investigated by comprehensive extracellular and intracellular metabolite profiling. The results were used to develop a partial-least-squares projection to latent structure regression (PLSR) prediction model and identify novel biomarkers for the different metastatic stages of melanoma.

## Results

### Metabolite and lipid profiling of human melanocytes and melanoma cells with different metastatic capacities

In this study, metabolomic and lipidomic analyses were simultaneously performed using polar and non-polar extracts of human epidermal melanocytes (HEMn-LP) and two melanoma cell lines (A375, A2058) with different stage of metastasis using GC-MS and DI-MS. As listed in Supplementary Table S1, a total of 39 metabolites were identified by GC-MS in HEMn-LP, A375, and A2058 cells: two alcohols (myo-inositol and glucitol), 19 amino acids (alanine, β-alanine, aspartic acid, cysteine, glutamine, glutamic acid, glycine, isoleucine, leucine, lysine, methionine, ornithine, proline, pyroglutamic acid, serine, threonine, tryptophan, tyrosine, and valine), five organic acids (aminomalonic acid, fumaric acid, lactic acid, malic acid, and succinic acid), four purines (hypoxanthine, inosine, guanine, and xanthine), two pyrimidines (uracil and uridine), five sugars (glucose, glyceric acid, glucose-6-phosphate, mannose-6-phosphate, and ribose), and other metabolites (creatinine, and phosphoric acid). The relative changes in these metabolites and significant differences between the three groups were determined by one-way analysis of variance (ANOVA) test. Among these metabolites, the levels of 10 amino acids, namely aspartic acid, cysteine, glutamic acid, glycine, lysine, methionine, ornithine, proline, pyroglutamic acid, and tyrosine, were increased in the two melanoma cell lines compared with the levels in normal cells, whereas those of four amino acids (alanine, leucine, serine, and threonine) were significantly decreased. The levels of all organic acids, sugars, and pyrimidines were also higher in low and high metastatic melanoma cells than in normal cells. In the case of purines, hypoxanthine was markedly upregulated in two melanoma cell lines relative to that in normal cells, whereas inosine and guanine were highest in the A2058 cell line.

In addition, comprehensive lipidomic profiling using nano-electrospray ionization (nanoESI) chip-based DI-MS was carried out on HEMn-LP, A375, and A2058 cells. To the best of our knowledge, this is the first study to investigate alterations in intact lipid species from melanoma cells with different metastatic properties using DI-MS. Representative averaged mass spectra of cells from pooled extracts are shown in Supplementary Fig. S1. The MS profiles of the spectra for an m/z range of 500–900 were found to be related to sphingolipids and glycerophospholipids. A total of 65 lipids, including nine phosphatidylcholine (PC), one plasmenyl-PC, and two each of plasmenyl-phosphatidylethanolamine (PE) and sphingomyelin (SM) species detected in the positive ion mode, and five ceramide (Cer), four plasmenyl-PE, eight PE, one cardiolipin (CL), seven phosphatidylglycerol (PG), 15 phosphatidylserine (PS), and 11 phosphatidylinositol (PI) species detected in the negative ion mode, were observed in melanocytes and two different melanoma cell lines. Detailed information on their fatty acyl chains, ion species, m/z values, and relative peak intensities is presented in Supplementary Tables S2 and S3.

The levels of plasmenyl-PE with 16:0/20:4 and plasmenyl-PE, PS, and PI species with 18:0/20:4 fatty acyl chains were significantly decreased in melanoma cells relative to the corresponding levels found in melanocytes. In contrast, the levels of plasmenyl-PE and PE species with other polyunsaturated fatty acyl chains, including C22:5 and C22:6 (plasmenyl-PE 16:0/22:5, 16:0/22:6, 18:0/22:5, PE 17:0/22:5, and 18:0/22:5), were significantly upregulated in the high metastatic melanoma group as compared to the normal and low metastatic groups, whereas these levels were unchanged or decreased in the low metastatic melanoma group compared to the normal group. Decreased levels of SM species, including d18:1/22:0 and d18:0/22:0, were detected in both melanoma groups relative to those in the normal group, and CL with four C18 and monounsaturated fatty acyl chains (CL 18:1/18:1/18:1/18:1) was lower in melanoma cells than in melanocytes. PI species, primarily, were increased with increases in metastatic potential, although some species were decreased in melanoma cells compared to those in normal cells. In particular, the levels of two PI species with saturated fatty acyl chains (16:0/18:0 and 18:0/18:0) were remarkably increased in melanoma cells relative to those in normal cells, with maximal levels observed in the most highly metastatic melanoma group. In contrast, unsaturated PI species such as PI 18:0/20:3 and 18:0/20:4 were substantially decreased in both melanoma cell lines.

### Prediction model and potential biomarkers for melanoma metastasis

**Figure 1:**
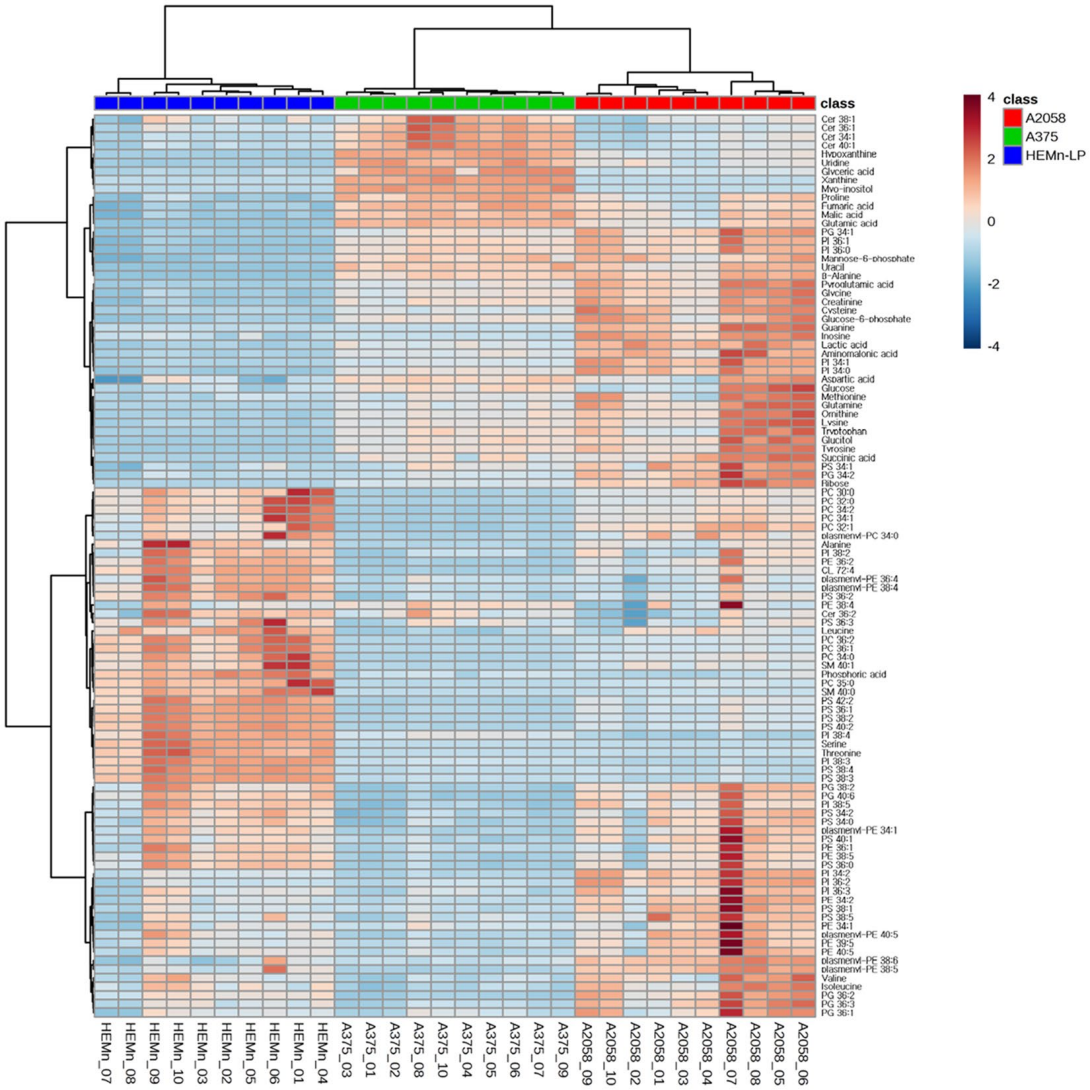
Heatmap representing the relative levels of metabolites and lipids in the three different cell types (HEMn-LP, A375, A2058).

A total of 104 identified metabolites and lipids, derived from three cell lines (HEMn-LP, A375, and A2058), were visualized by heatmap with hierarchical clustering (Fig. 1). The results of a hierarchical clustering analysis of the heatmap showed that the normal, low metastatic, and high metastatic groups, represented by blue, green, and red, respectively, clustered separately. This separation suggests that the profiles of metabolites and lipids are highly distinct among the three groups with different metastatic properties. The heatmap also showed changes in the relative concentration of each metabolite and lipid by colour scheme. These metabolic patterns provide an overview of the metabolic differences between the three cell groups, but a more detailed analysis was required to better understand metabolism related to melanoma metastasis.

**Figure 2:**
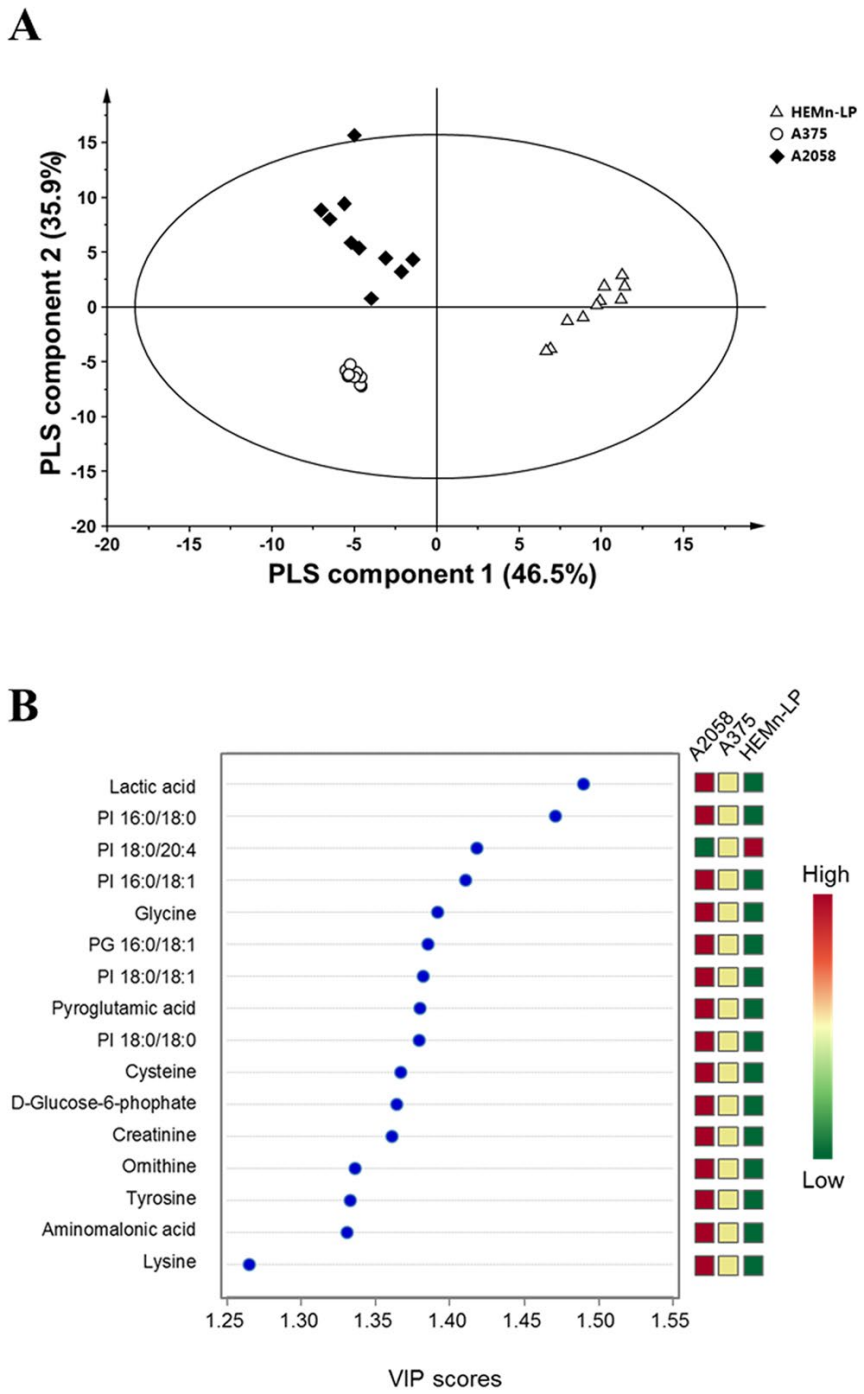
PLS-DA analysis based on metabolic and lipidomic data. (**A**) PLS-DA-derived score plot from melanocytes and two melanoma cell lines (*n* = 10 for each group) and (**B**) important metabolites and lipids selected based on VIP scores.

For this purpose, a partial least-squares (PLS)-based multivariate classification method, partial least-squares discriminant analysis (PLS-DA), was employed to compare the three groups of normal cell and melanoma cells with low and high metastatic potential, based on identified metabolite and lipid profiling datasets. As shown in Fig. 2A, the PLS-DA derived score plot revealed that the normal and melanoma groups were clearly separated by PLS component 1, which explained 46.5% of the variance. Furthermore, the low metastatic and high metastatic melanoma groups were separated by PLS component 2, which explained 35.9% of the variance. These results imply that various changes in metabolic and lipidomic profiles occur in association with the metastatic properties of melanoma cell lines. Permutation tests were randomly performed to validate the PLS-DA model. In general, an *R* ^2^ *Y* intercept < 0.3–0.4 and *Q* ^2^ *Y* intercept < 0.05 indicate a valid model^30^, and this model showed acceptable values for the *R* ^2^ *Y* intercept of 0.181 and *Q* ^2^ *Y* intercept of −0.466.

**Figure 3:**
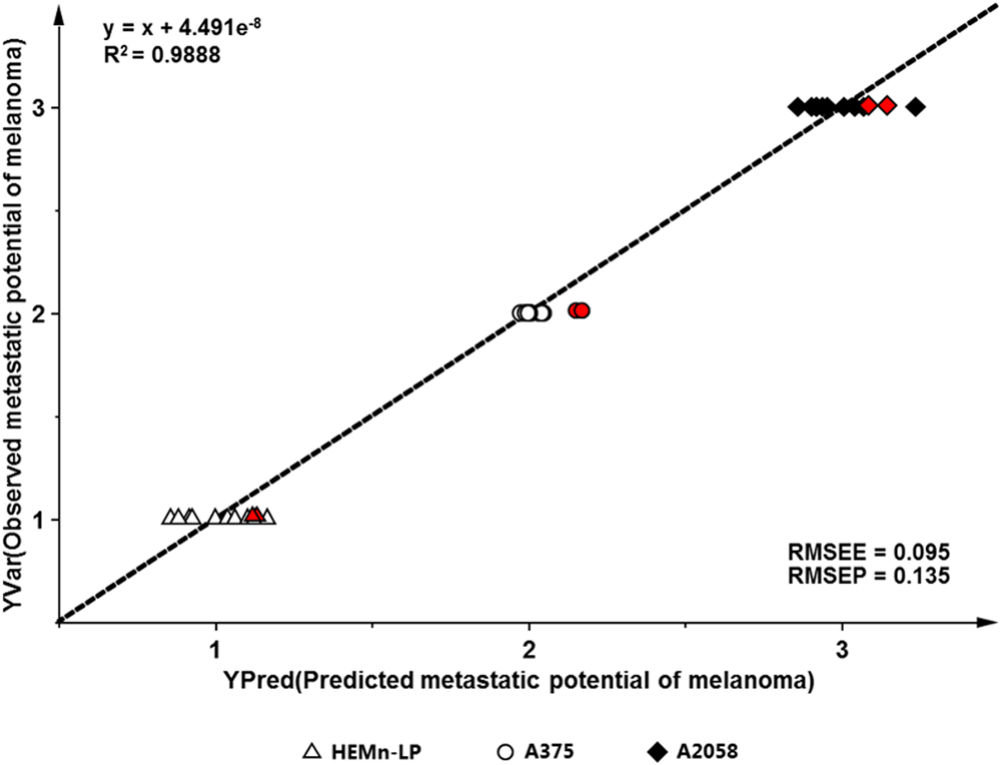
Establishment and validation of the PLSR model for prediction of the metastatic stage of melanocyte and metastatic melanoma cells (calibration dataset, *n* = 10, black and white; validation dataset, *n* = 2, red).

Additionally, PLS regression was implemented to determine the correlation between the metabolome and lipidome profiles and metastasis of melanoma. The relationship between the observed and predicted metastatic potential of melanoma cells determined by the PLS regression model developed from the training dataset containing 104 variables of 10 individual samples of each of HEMn-LP, A375 and A2058 cells (Fig. 3). The dataset was generated from experimental data that are the relative intensities of identified metabolites and lipids from each group. The regression equation of the PLSR model from the training set was expressed as y = x + 4.491e^−8^ with a high correlation coefficient value (*R* ^2^ = 0.9888), and the model yielded high *R* ^2^ *Y* and *Q* ^2^ *Y* values of 0.989 and 0.975, respectively. The values of *R* ^2^ *Y* and *Q* ^2^ *Y* are used to estimate the reasonable fit and predictability of the model, and they vary between 0 and 1. When *Q* ^2^ *Y* is higher than 0.5, the prediction model is regarded as good, and if the value is higher than 0.9, it is considered excellent^30^. Subsequently, an independent dataset from each group was imported into the PLS calibration plot for external validation, and these data fit perfectly into the regression line obtained using the calibration data. The root-mean-square error of estimation (RMSEE) and root-mean-square error of prediction (RMSEP) were also used to evaluate the quality of the PLSR model. In this model, low validation error values (an RMSEE of 0.095 and RMSEP of 0.135) was achieved. These results suggest that this model can be used as practical tool to predict metastatic potential in patients with melanoma.

Metabolites and lipids with variable influence on projection (VIP) values > 1.0, which strongly contributed to the ability to discriminate between the three cell lines in the PLS-DA model, were first selected as biomarker candidates. Among them, 16 important molecules with levels that were significantly and progressively increased or decreased with the increasing metastatic potential of melanoma cells were selected as potential biomarkers for the prognosis of metastatic melanoma. These biomarkers are described in Fig. 2B. Of the 39 metabolites identified by GC-MS, 10 metabolites, including six amino acids, two organic acids, one sugar, and creatinine, were selected as potential biomarker candidates for melanoma, and the cellular levels of these metabolites showed progressive elevation with advancing stages of melanoma. Among a total of 65 lipids assigned by DI-MS, five PI species (16:0/18:0, 16:0/18:1, 18:0/18:0, 18:0/18:1, and 18:0/20:4) and one PG species (16:0/18:1) were identified as lipid biomarker candidates to distinguish between melanomas with low and high metastatic potential, as well as to distinguish between malignant melanoma cells and normal ones. The levels of lipid biomarkers, except for that of PI 18:0/20:4, were highest in the high metastatic cells, followed by low metastatic and normal cells.

**Figure 4:**
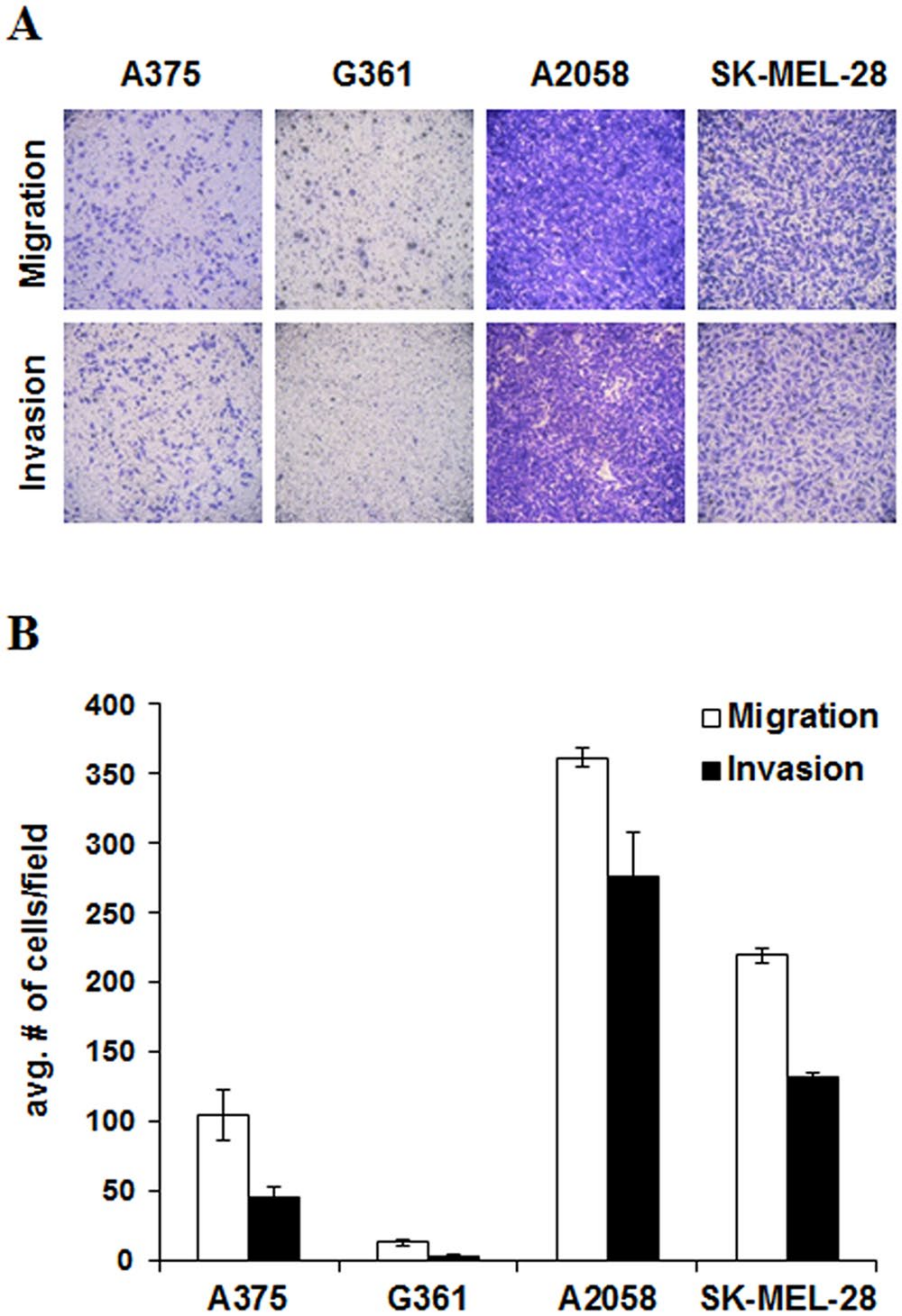
The migratory and invasive potential of the melanoma cell lines (A375, G361, A2058, and SK-MEL-28). (**A**) Represented images of migrated and invaded cells and (**B**) quantified data (mean ± SEM) from three independent experiments.

**Figure 5:**
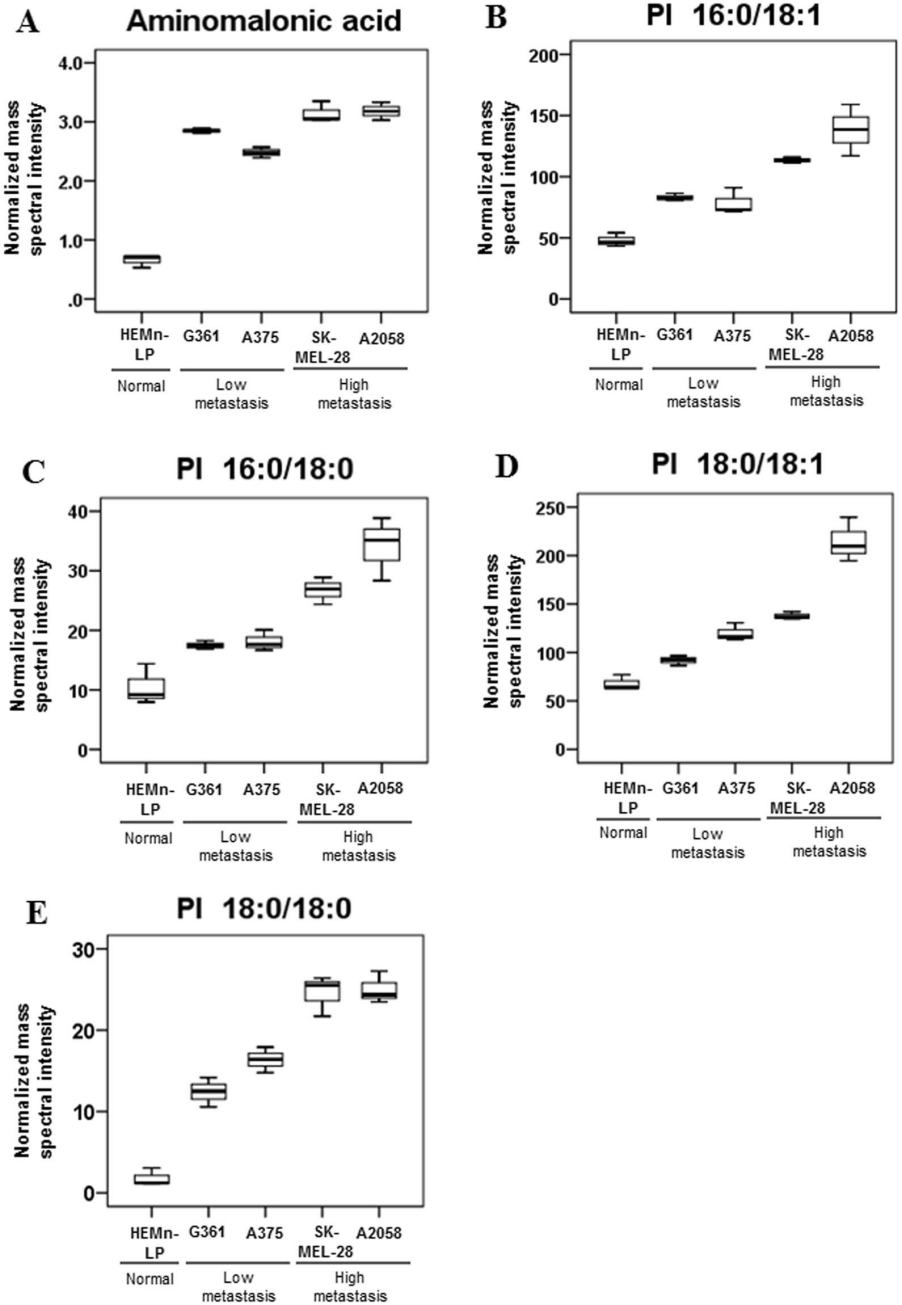
Box plots depicting the relative levels of potential biomarkers, (**A**) aminomalonic acid, (**B**) PI 16:0/18:1, (**C**) PI 16:0/18:0, (**D**) PI 18:0/18:1 and (**E**) PI 18:0/18:0 of normal human melanocytes and melanoma cells with different metastatic potential. Normal human melanocyte (HEMn-Lp), low metastatic melanoma cell lines (A375, G361), and highly metastatic melanoma cell lines (A2058, SK-MEL-28).

To verify those potential biomarkers for predicting metastatic stages of melanoma, metabolite and lipid profiling were performed using two additional melanoma cell lines (G361, SK-MEL-28) with different metastatic potentials. In addition, we assessed the migratory and invasive potentials of A375, A2058, G361, and SK-MEL-28 melanoma cell lines. Migration and invasion assays demonstrated that A375 and G361 had lower migratory and invasive capability than A2058 and SK-MEL-28 cells (Fig. 4). As shown in Fig. 5, higher relative levels of aminomalonic acid, PI 16:0/18:1, PI 16:0/18:0, PI 18:0/18:1, and PI 18:0/18:0 were observed in low metastatic cell lines relative to normal cell line. The levels of those were also higher in highly metastatic cell lines relative to low metastatic cell lines. From those findings, those five compounds were identified as novel potential biomarkers which can differentiate the melanoma according to different metastatic potentials. The fragmentation mass spectra of the identified biomarkers of melanoma are presented in supplementary Fig. S2.

### Pathway analysis based on metastasis-associated metabolomic and lipidomic alterations in melanoma

**Figure 6:**
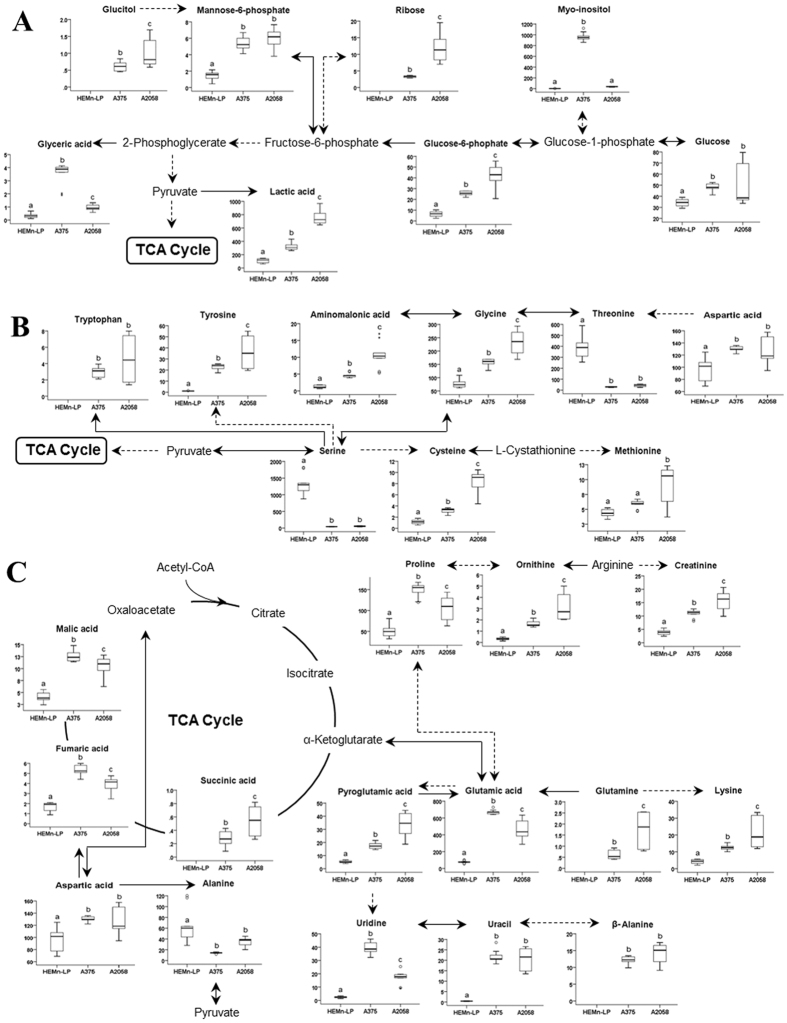
Metabolic changes and associated pathways in melanocytes and melanoma cells with different metastatic potential (HEMn-LP, A375, A2058), as determined by gas chromatography-mass spectrometry. Major pathways associated with melanoma metastasis, (**A**) glycolysis, (**B**) glycine, serine and threonine metabolism, and cysteine and methionine metabolism, (**C**) alanine, aspartate and glutamate metabolism, arginine and proline metabolism, and β-alanine metabolism were identified. Metabolic pathways were proposed based on a comparison with data in the KEGG database (http://www.genome.jp/kegg/). ANOVA, followed by Tukey’s post-hoc test (*p* < 0.05), was conducted, and different letters indicate statistically significant differences between samples.

**Figure 7:**
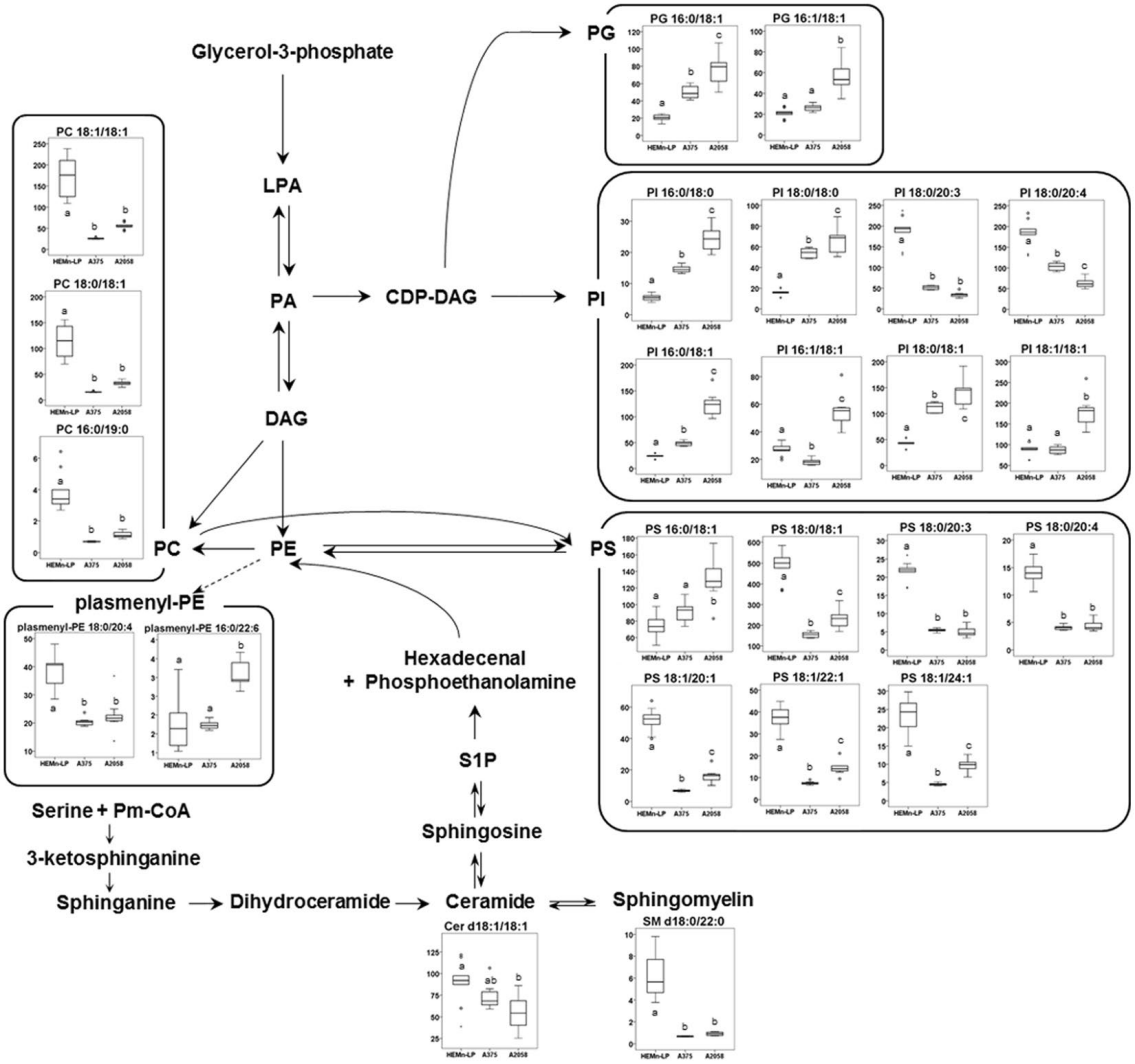
Schematic representation of the glycerophospholipid and sphingolipid pathways in human melanoma cells. Box plots show relative changes in lipid species with VIP > 1.0 in melanocytes and melanoma cells with different metastatic potential (HEMn-LP, A375, A2058) as determined by direct infusion-mass spectrometry. ANOVA, followed by Tukey’s post-hoc test (*p* < 0.05), was conducted, and different letters indicate statistically significant differences between samples. Proposed pathways were based on data in the KEGG database (http://www.genome.jp/kegg/).﻿ PC, phosphatidylcholine; PE, phosphatidylethanolamine; PG, phosphatidylglycerol; PI, phosphatidylinositol; PS, phosphatidylserine; LPA, lysophosphatidic acid; PA, phosphatidic acid; DAG, diacylglycerol; CDP-DAG, cytidine diphosphate-diacylglycerol; Pm-CoA, palmitoyl coenzyme A; S1P, sphingosine-1-phosphate﻿.

To identify major metabolic pathways that are altered with melanoma metastasis, the 39 identified metabolites were uploaded into the web-based tool for pathway analysis, MetaboAnalyst 3.0 (http://www.metaboanalyst.ca). The top seven ranked metabolic pathways with high impact scores and statistical significance cutoffs of *p* < 0.05 were associated with the development of melanoma (Table 1). The impact score threshold based on pathway topological importance was set at 0.10, and seven metabolic pathways in the three different cell types were identified as relevant pathways in terms of metabolic perturbation. Alanine, aspartate and glutamate metabolism showed the highest impact score (0.71), and glycine, serine and threonine metabolism, and arginine and proline metabolism were also significantly altered in conjunction with melanoma metastasis. In addition, β-alanine metabolism, aminoacyl-tRNA biosynthesis, cysteine and methionine metabolism, and d-glutamine and d-glutamate metabolism were associated with the metastatic process. Details of metabolic pathways with differences between the control and melanoma groups are shown in Fig. 6. To understand of lipid metabolism that also contributes to cellular processes, including metastasis, proliferation, and invasion, the main altered lipid pathways for glycerophospholipid and sphingolipid biosynthesis were investigated (Fig. 7). Our data showed that lipid metabolism was perturbed with different metastatic properties of melanoma, and, in particular, that most PI and PG species increased with increasing metastatic potential, whereas PC, PS, and SM species decreased.

**Table 1:** List of metabolic pathways associated with melanoma metastasis.

No.	Pathway name	Total	Hits	*p*	−log(*p*)	Impact
1	Alanine, aspartate and glutamate metabolism	24	6	1.32E-06	13.54	0.71
2	Glycine, serine and threonine metabolism	48	7	7.59E-06	11.79	0.42
3	Arginine and proline metabolism	77	7	1.76E-04	8.65	0.27
4	β-Alanine metabolism	28	3	9.74E-03	4.63	0.26
5	Aminoacyl-tRNA biosynthesis	75	16	3.25E-15	33.36	0.23
6	Cysteine and methionine metabolism	56	5	1.79E-03	6.33	0.18
7	d-Glutamine and d-glutamate metabolism	11	2	1.28E-02	4.36	0.14

Pathways were identified by a pathway analysis using MetaboAnalyst.

## Discussion

This study describes metabolic changes related to melanoma metastasis using GC-MS. Accumulation of organic acids was found in two melanoma cell lines compared to the levels in melanocytes, and, in particular, lactic acid, the end product of glycolysis, increased progressively with the metastatic potential of melanoma cells. This increase was associated with an elevation in LDH, which converts pyruvate to lactic acid, in the blood of patients with melanoma, representing an important indicator of metastases in malignant melanoma^5^. Most cancer cells utilize glycolysis predominantly rather than mitochondrial oxidative phosphorylation for energy production, and it accelerates glucose uptake and production of lactic acid^31, 32^. In a previous study, elevated glucose transporter isoform 1 (GLUT1) expression was shown to promote glucose uptake and cell progression in primary and metastatic melanoma tissues compared to those in benign nevi^33^. Furthermore, increased glucose uptake was also linked to an increased level of glucose-6-phosphate in the glycolytic pathway, and highly accumulated glucose-6-phosphate was found in high metastatic melanoma cells in our study.

Among the organic acids, aminomalonic acid was identified as a potential biomarker of melanoma metastasis for the first time. The level of aminomalonic acid was significantly elevated in low and high metastatic melanoma cells when compared to normal cells, which is consistent with previous reports of an increased level of aminomalonic acid in lung, hepatocellular, and colorectal cancers compared to that of normal controls^13, 34, 35^. The origin of aminomalonic acid was suggested under defect in protein synthesis and free radical damage to proteins^36^. Additionally, this metabolite was found to be generated by serine hydroxylmethyltransferase (SHMT), the enzyme catalyzing glycine carboxylation that was reported as a potential therapeutic target for cancer^36, 37^. Overexpression of SHMT1 was also identified in lung metastases from breast cancer patients, and SHMT1 was suggested to be a prognostic factor for shorter overall survival for patients with metastatic breast cancer^38^.

Metabolic changes were also observed in amino acids, including glycine, pyroglutamic acid, cysteine, ornithine, tyrosine, and lysine which showed a progressive increase with the increasing metastatic potential of cells. Previous studies have shown that the levels of amino acids to synthesize proteins and nucleic acids were higher in various tumor tissues, including lung, colon, and stomach cancers, relative to those in normal tissues^13, 39^. The main source of amino acids is degraded products from the extracellular matrix, especially those degraded by matrix metalloproteinases (MMPs), and uptake of amino acids is mediated by amino acid transporters, which are highly upregulated in cancer cells to meet the high demand for amino acids to support cell growth, survival, and proliferation^39^.

Of all the major metabolic pathways involved in melanoma metastasis, alanine, aspartate and glutamate metabolism was the top affected pathway with the highest impact score. During cell proliferation, alanine is used for protein synthesis, and it is secreted as a glycolytic byproduct to transport excess carbon from glycolysis^40^. Based on metabolic profiling of extracellular metabolites in culture medium, the levels of alanine secreted by A2058 were higher than by A375 melanoma cells (Supplementary Fig. S3). Aspartate is converted to oxaloacetate, which is a tricarboxylic acid (TCA) cycle intermediate, by aspartate aminotransferase and contributes to electron transfer reactions, as well as protein and nucleotide synthesis^40^. Expression of the c-myc oncogene was increased in metastatic melanoma compared to primary melanoma and nevi, and myc promoted glutamine uptake and glutaminolysis^41, 42^. This metabolism is an important source of energy production in cancer cells, and TCA cycle intermediates such as α-ketoglutarate, fumarate, malate, succinate, and glutamate were increased by glutamine degradation^41^, which is consistent with our results. Elevated pyroglutamic acid, a reservoir of glutamate^43^, was identified in highly metastatic melanoma cells, and release of this metabolite into medium also increased in highly metastatic A2058 compared to that in low metastatic A375 cells. Additionally, in our study, ornithine was found to be belonged to arginine and proline metabolism, and both intracellular and extracellular levels of this metabolite were higher in A2058 cells than in A375 melanoma cells.

Based on lipidomic profiling, the levels of lipid species with 20:4 fatty acyl chains such as plasmenyl-PE 16:0/20:4, plasmenyl-PE 18:0/20:4, PS 18:0/20:4, and PI 18:0/20:4 were decreased in melanoma cells compared to those in normal cells. Arachidonic acid (C20:4) is an important prostaglandin precursor that is released from cell membrane glycerophospholipid by phospholipase A_2_ and is converted into prostaglandin E_2_ (PGE_2_) by cyclooxygenase-2 (COX-2) and PGE synthase^44, 45^. Abnormally increased levels of COX-2 and PGE_2_ play important roles in the development, progression, and metastasis of numerous cancers, including prostate cancer, colorectal cancer, and melanoma^46–49^. This implies that decreased levels of lipid species with C20:4 are associated with increases in PGE_2_ derived from free arachidonic acid, which contributes to the development and metastasis of melanoma cells.

SMs are the major components of cellular membranes and are important molecules in the regulation of functions such as survival, proliferation, and migration of cancer cells^50^. Recently, it was reported that deficiency of SM synthase enhanced cell migration by modulating the response to cell motility through a CXCL12/CXCR4 signaling pathway in an SMS1/SMS2 double-knockout cellular model^51^. Therefore, in our results, the decreased levels of SM in melanoma cells compared to those in normal cells appear to be closely related with melanoma progression and migration. The effects of exogenous sphingomyelin on migration of melanoma should be investigated in the future.

CL is an anionic phospholipid located exclusively in the mitochondrial membrane that plays essential roles in maintaining mitochondrial function and membrane integrity. Abnormalities in the content or composition of CL have been found in cancer cells^52^. For example, decreased CL species were found in mitochondria isolated from nonmetastatic and metastatic mouse brain tumors relative to those in normal tissues by shotgun lipidomics. These observations are consistent with our results in melanoma cells. CL deficiency has been suggested to reduce electron transport chain activity and impair respiratory function in cancer cells^53^.

PI species containing saturated fatty acyl chains (16:0/18:0, 18:0/18:0) increased progressively in cells with higher metastatic potential. A previous lipidomic study showed that the ratio of saturated to unsaturated fatty acids increased in the plasma membranes of highly metastatic B16-F10 cells as compared to that in low metastatic B16-F1 cells, suggesting that membrane fluidity is decreased in highly metastatic melanoma by promoting saturated fatty acids in the plasma membrane^22^. Phospholipid saturation protects cancer cells from endogenous and exogenous damage such as lipid peroxidation and chemotherapeutic agents and affects signal transduction^54^. PI species are the precursors of phosphatidylinositol-3,4,5-trisphosphate (PIP_3_), a signaling lipid that regulates cell growth, proliferation, and migration, and increases in PIP_3_ promote the transition to malignancy^55, 56^. PIP_3_ generation is promoted by phosphatidylinositol-3-kinase (PI3K), which phosphorylates the inositol ring of phosphatidylinositol lipids and plays an important role in melanoma progression. Previously, overexpression of the p85 and p110 subunits of PI3K was observed in malignant melanoma tissues relative to benign tissues. In particular, p85 expression was higher in metastatic melanomas relative to primary melanomas in a large cohort study^57^. PIP_3_, the product of PI3K, serves as a docking site to recruit signaling proteins with pleckstrin homology domains including Akt and PDKs to the cellular membrane, where Akt is activated by PDK1 and PDK2^58^. Activated Akt has been shown to suppress transcription of the cell adhesion molecule E-cadherin, thereby leading to increased motility and a more invasive phenotype of melanoma cells^59–61^. In addition, PI3K/Akt signaling regulates angiogenesis, which is essential for tumor growth and metastasis. Expression of angiogenetic cytokines such as vascular endothelial growth factors (VEGFs) is increased by hypoxia-inducible factor 1 (HIF-1) through activation of PI3K/Akt and was found to be higher in advanced melanoma tissue than benign nevi^62, 63^. *p*-Akt was also suggested as prognostic biomarker for patients with melanoma, whose increased expression was significantly related with melanoma invasion and a poorer 5-year patient survival in a clinicopathologic study of 292 cases: normal nevi, dysplastic nevi, primary melanomas, and metastatic melanomas^64^. Based on those findings, the accumulation of PI species, including PI 16:0/18:0, 16:0/18:1, 18:0/18:0, and 18:0/18:1, was shown to be closely associated with melanoma metastasis and progression, and these lipid species can be considered novel biomarkers for estimating the metastatic ability of melanoma cells.

Accumulation of PI species provides melanoma cells with key signaling molecules that enhance metastasis of melanoma via PI3K/Akt signaling; therefore, components of the PI biosynthetic pathway may offer new targets for therapeutic intervention in melanoma. PI synthesis can occur via an enzymatic cascade involving two major enzymes: cytidine diphosphate-diacylglycerol synthetase (CDS) and phosphatidylinositol synthase (PIS). PIS is responsible for the last step in biosynthesis of PI in the endoplasmic reticulum (ER), catalyzing the formation of PI and cytidine monophosphate (CMP) from cytidine diphosphate-diacylglycerol and *myo*-inositol^65, 66^. Inhibition of PIS may prevent the production of precursors for signaling lipids, including PIs, which play crucial roles in survival, proliferation, and metastasis of cancer cells. Previous studies have shown that expression of PIS protein is increased in human head and neck squamous carcinoma cell lines and biopsy specimens from oral squamous cell carcinoma compared to normal cells and tissues, and inhibition of PIS induced G1 arrest in the cell cycle of oral cancer cells by reducing cellular levels of cyclin D1, cyclin E, and phosphorylated Prb^67, 68^. In addition, inostamycin, an inhibitor of PIS, was reported to suppress the invasion ability of tongue carcinoma cells by reducing MMP2 and −9 production^69, 70^. PIS is an enzyme specific to the PI synthesis pathway; however, CDS is also essential to the PI synthesis pathway. CDS that is located on the cytoplasmic side of the ER regulates the biosynthesis of PI, and, on the matrix side of the mitochondrial inner membrane, it synthesizes the precursor of PG^71, 72^. In this study, PG as well as PI selected as biomarker candidates were increased with increases in the metastatic potential of melanoma cells relative to those of the control. Therefore, inhibition of CDS activity may be a target in the treatment of melanoma.

In this study, we found that alterations in the metabolite and lipid profiles of human melanocytes and melanoma cell lines were related to cancer metastasis potential and demonstrated a PLSR model for predicting metastatic potential of melanoma. The PLSR model was established by using multiple variables (104 variables), and the prediction model has high accuracy compared to univariate methods based on changes in the level of single molecule. To our knowledge, this is the first study to investigate therapeutic targets for the development of novel antimetastatic drugs, as well as potential biomarkers to predict the metastatic potential of melanoma through metabolic and lipidomic analyses. We propose, for the first time, the use of aminomalonic acid, and PI species with saturated and monounsaturated fatty acyl chains (PI 16:0/18:0, 16:0/18:1, 18:0/18:0, and 18:0/18:1) as potential novel biomarkers for melanoma metastasis, as well as the potential for targeting metabolic and lipidomic pathways for cancer therapies. Implementation of this experimental design on clinical tissue or plasma levels of melanoma patients will be needed to verify those biomarkers in the future. The results of this study may lead to the development of tools to diagnose or modulate the metastasis of melanoma.

## Methods

### Chemicals and reagents

High-performance liquid chromatography (HPLC)-grade methanol, chloroform, and water were purchased from Fisher Scientific (Pittsburg, PA). HPLC-grade hexane was purchased from Honeywell Burdick & Jackson (Muskegon, MI). Butylated hydroxytoluene (BHT), myristic-d_27_ acid, methoxylamine hydrochloride, pyridine, and ammonium acetate were purchased from Sigma-Aldrich (St. Louis, MO). BSTFA [*N,O*-bis(trimethylsilyl) trifluoroacetamide] containing 1% trimethylchlorosilane (TMCS) was purchased from Alfa Aesar (Ward Hill, MA).

### Cell culture and sample collection

Primary (A375, G361) and metastatic (A2058, SK-MEL-28) human melanoma cell lines, characterized as having low and high metastatic potential (American Type Culture Collection, Manassas, VA; Korean Cell Line Bank, Seoul, South Korea), were cultured in Dulbecco’s modified Eagle’s medium (A375, A2058), Roswell Park Memorial Institute 1640 medium (G361) and minimum essential medium (SK-MEL-28) supplemented with 10% fetal bovine serum and 1% penicillin-streptomycin (Hyclone Labs, Logan, UT). Human epidermal melanocytes (HEMn-LP cells) were cultured in 254 medium containing human melanocyte growth supplement (HMGS). All cell lines were cultured in a humidified incubator with 5% CO_2_ at 37 °C. The cells were sub-cultured at a seeding density of 1 × 10^6^ cells/mL and were grown to approximately 90% confluence for metabolic and lipidomic profiling experiments. Cells from each line were harvested by treatment with trypsin- ethylenediaminetetraacetic acid (EDTA), and lysed by two freeze-thaw cycles. Then, protein concentrations were determined using a Bio-Rad Protein Assay Kit (Thermo Scientific, Rockford, IL) with bovine serum albumin (BSA) standards for normalization. The cells were dried in a freeze dryer (IlShinBioBase, Dongduchun city, Kyunggi-do, Korea) and stored at −70 °C until analysis. Additionally, media samples were collected before (fresh) and after (spent) 2–3 days of cell culture and stored at −70 °C for later extracellular metabolite profiling.

### Cell migration and invasion assays

Cells were plated in the upper chamber of an insert (Corning-Costar, Lowell, MA, USA) coated with or without collagen 1 (Sigma-Aldrich, St. Louis, MO, USA) containing 1% FBS-media for cell invasion or cell migration assays, respectively. The bottom chamber contained 10% FBS-media as a chemoattractant. After 8 to 10 h of incubation, cells and medium were removed from the upper chamber and migrated or invaded cells were fixed with methanol and stained with 0.5% crystal violet (Sigma-Aldrich, St. Louis, MO, USA). Images were taken at ×100 magnification under a bright-field microscope (Olympus CKX41, Tokyo, Japan). Quantification was performed under a light microscope with × 200 magnification by counting migrated or invaded cell number in five random fields per chamber.

### Extraction of metabolites and lipids from cell

Metabolites and lipids were extracted from freeze-dried cells using a modified Folch procedure^73^. Briefly, 1 mL ice-cold chloroform and 0.5 mL methanol, with 0.1% BHT were added to the freeze-dried cells and vortexed for 20 sec. Then, the mixture was sonicated for 30 min in a 4 °C water bath and incubated for 60 min with shaking on ice. Phase separation was induced by adding 0.38 mL of ice-cold water with 0.1% BHT, followed by incubation for 10 min with shaking on ice. The mixture was centrifuged at 18,500 × g for 10 min at 4 °C, then split into two aliquots, the upper phase for metabolic profiling by GC-MS, and the lower phase for lipid profiling by DI-MS. The extracts were passed through 0.2 μm PTFE syringe filter (Whatman, Maidstone, UK), and then dried with nitrogen gas. The dried methanol fraction was used for derivatization procedures, and the dried chloroform fraction was reconstituted in 100 μL of methanol: chloroform (9:1, v/v) containing 7.5 mM ammonium acetate buffer solution for lipid analysis.

### Extraction of metabolites from cell culture medium

Culture medium samples were thawed in a 37 °C water bath for 20 min, and 0.5 mL of medium was transferred to a centrifugal filter device and centrifuged at 18,472 × g for 15 min at 4 °C. After centrifugation, 0.2 mL of supernatant was transferred to a glass vial and 0.8 mL of 100% methanol was added. The samples were briefly vortexed and sonicated for 30 min. Then, 0.2 mL of the resulting medium extract was transferred to a gas chromatography (GC) vial and evaporated with nitrogen gas for GC-MS analysis.

### GC-MS analysis

For derivatization, 0.4 mL of each cell extract and 0.2 mL of each medium extract was transferred to a GC vial and dried with nitrogen gas flow, 30 μL of 20,000 μg/mL methoxylamine hydrochloride in pyridine was used for oximation, and then 50 μL of *N,O*-bis(trimethylsilyl) trifluoroacetamide (BSTFA) containing 1% TMCS, and 10 μL of myristic-d_27_ acid in pyridine (500 μg/mL as an internal standard) were added to the dried samples. The samples were incubated for 60 min at 65 °C before GC-MS analysis.

GC-MS analysis was performed on an Agilent 7890 A gas chromatograph (Agilent Technologies, CA) with an autosampler (7683B series, Agilent Technologies) and a 5975 C mass selective detector (Agilent Technologies, CA). A DB5-MS column (a fused silica capillary column of 5% phenylmethylpolysiloxane phase with a 30 m length × 0.25 mm internal diameter × 0.25 μm film thickness, Agilent Technologies) was used for analysis, and 1.0 μL of each sample was injected into a split/splitless inlet at 250 °C. The carrier gas used was helium at a constant flow rate of 1.0 mL/min. In electron impact ionization mode, an electron energy of 70 eV and a split ratio of 1:10 were used. The temperature of the ion source, quadrupole, and auxiliary were set at 230, 150, and 280 °C, respectively. The mass range was 50–700 Da, and the data were gathered in full scan mode. For the analysis of metabolites in cell samples, the initial oven temperature was set at 70 °C and then programmed to increase to 190 °C (at 5 °C/min), 240 °C (6 °C/min), and finally 280 °C (5 °C/min). In addition, for the medium analysis, the initial oven temperature was 70 °C and increased to 300 °C (at 5 °C/min). All metabolites of cells and medium were identified by comparison with data in the Human Metabolome Database (HMDB; http://www.hmdb.ca/), Golm Metabolome Database (GMD; gmd.mpimp-golm.mpg.de/), and NIST-Wiley Mass Spectra Library.

### Lipid analysis

Lipids were analyzed using a linear ion-trap mass spectrometer (LTQ-XL, Thermo Fisher Scientific, San Jose, CA) equipped with an automated nanoinfusion/nanospray source (TriVersa NanoMate System, Advion Biosciences, Ithaca, NY) in positive and negative ion modes. Ten microliters of lipid extract was aspirated and infused into the MS system through a nanoelectorospray chip containing 5.5-µm diameter spray nozzles. The ion source was controlled and manipulated using Chipsoft 8.3.1 software (Advion Biosciences). Ionization voltage and gas pressure were set to 1.4 kV and 0.4 psi, respectively, in positive ion mode, and to 1.7 kV and 0.6 psi in negative ion mode. The mass spectrometer settings included capillary voltages of 29 V (positive ion mode) and −33 V (negative ion mode), tube lens voltages of 145 V (positive ion mode) and −83 V (negative ion mode), and a capillary temperature of 200 °C. Each sample was analyzed in profile mode for 2 min, and the scan range was set at m/z 400–1,200 for both the positive and negative ion modes. Tandem mass spectrometry (MS/MS) was applied to samples pooled from each group to identify lipid species. MS/MS conditions included a normalized collision energy of 35%, an isolated width of 1.5 m/z units, and a charge state of 1. The dynamic exclusion parameters were a repeat duration of 60 sec, exclusion duration of 60 sec, and exclusion list size of 50. Lipid species were identified using databases of Lipidmaps (http://www.lipidmaps.org/), LipidBlast by Kind *et al*.^74^, and an in-house MS/MS library. In addition, the identification of ceramide species was based on authentic reference MS/MS spectra by Han^75^.

### Spectral data processing

The raw data files (*.raw) were converted to *.mzXML format in Proteo Wizard MSConvert^76^, then mass spectra were further processed using Expressionist® MSX software (version 2013.0.39, Genedata, Basel, Switzerland). The data were normalized by dividing by the peak intensity of the internal standard and total protein content.

### Statistical analysis

The resulting datasets, maintained in Microsoft Office Excel (version 2010; Microsoft, Redmond, WA), were used for Student’s *t*-test (at a threshold of *p* < 0.05), cluster analysis (heatmap), and pathway analysis in the web-based software tool MetaboAnalyst (version 3.0; http://www.metaboanalyst.ca). Significant differences in the levels of each metabolite were determined by one-way ANOVA followed by Tukey’s post-hoc test using SPSS software (version 23, IBM, Somers, NY). For the multivariate statistical analysis, all data were mean centered and scaled to unit variance, and the PLS-DA and PLSR were performed using SIMCA-P+ software (version 13.0, Umetrics, Umeå, Sweden).

## Electronic supplementary material

Supplementary Materials
