# Supplementary material for: Discovery of potential biomarkers in human melanoma cells with different metastatic potential by metabolic and lipidomic profiling
Source: Sci Rep. 2017 Aug 18;7:8864. doi: 10.1038/s41598-017-08433-9 (PMC5562697; doi:10.1038/s41598-017-08433-9)
Supplement: Supplementary file 1 — Supplementary Materials [file 41598_2017_8433_MOESM1_ESM.pdf]

Supplementary Materials

Discovery of potential biomarkers in human melanoma cells with different metastatic potential by metabolic and lipidomic profiling

Hye-Youn Kim, Hwanhui Lee, So-Hyun Kim, Hanyong Jin, Jeehyeon Bae, Hyung-Kyoon Choi*

College of Pharmacy, Chung-Ang University, Seoul 06974, Republic of Korea

*To whom correspondence should be addressed

Tel: +82-2-820-5605

Fax: +82-2-812-3912

E-mail: hykychoi@cau.ac.kr

Supplementary Table S1. Global metabolic profiles of human primary melanocytes (HEMn-LP) and melanoma cell lines (A375, A2058) using GC-MS. ANOVA was performed to detect statistically significant differences between samples ($p < 0.05$). Different letters indicate statistically significant differences between metabolite levels. RT, retention time; TMS, trimethylsilylation; ND, not detected.

No.	Compound	RT (min)	Fragmentation ion (m/z)	TMS	HEMn-LP	A375	A2058
	**Alcohol**						
1	Myo-inositol	28.93	191, 217, **305**, 318	6	1.29 ± 0.42[a]	964.61 ± 75.51[b]	35.38 ± 7.50[a]
2	Glucitol	26.27	103, 205, 217, **319**	6	ND	0.62 ± 0.14[b]	1.02 ± 0.42[c]
	**Amino acid**						
3	Alanine	7.01	100, **116**, 190, 218	2	63.39 ± 31.37[a]	14.09 ± 1.14[b]	34.51 ± 8.45[b]
4	β-Alanine	15.13	133, **174**, 248, 290	3	ND	12.03 ± 1.26[b]	13.97 ± 2.90[b]
5	Aspartic acid	17.43	100, 202, 218, **232**	3	96.47 ± 20.16[a]	129.69 ± 4.65[b]	127.16 ± 22.50[b]
6	Cysteine	18.22	59, 100, 132, **220**	3	1.14 ± 0.39[a]	3.14 ± 0.48[b]	8.13 ± 2.15[c]
7	Glutamine	23.12	**156**, 203, 245, 347	3	ND	0.60 ± 0.20[b]	1.68 ± 0.76[c]
8	Glutamic acid	19.78	128, 156, 218, **246**	3	76.16 ± 14.04[a]	669.94 ± 25.81[b]	458.06 ± 116.12[c]
9	Glycine	7.47	59, **102**, 176, 204	2	78.19 ± 15.56[a]	156.19 ± 15.15[b]	230.97 ± 44.23[c]
		12.03	86, 100, **174**, 248	3			
10	Isoleucine	11.73	100, 117, **158**, 218	2	26.03 ± 6.86[a]	15.24 ± 2.95[b]	35.00 ± 10.04[c]
11	Leucine	8.31	75, **86**, 146, 188	1	6.56 ± 1.48[a]	2.20 ± 0.65[b]	3.95 ± 1.23[c]
12	Lysine	26.08	128, 156, **174**, 317	4	4.23 ± 1.25[a]	12.54 ± 1.72[b]	21.59 ± 9.23[c]
13	Methionine	17.34	61, 128, 156, **176**	2	4.34 ± 0.65[a]	5.89 ± 0.67[a]	8.91 ± 3.25[b]
14	Ornithine	23.99	**142**, 174, 200, 420	4	0.32 ± 0.11[a]	1.63 ± 0.24[b]	3.16 ± 1.17[c]
15	Proline	11.81	100, 133, **142**, 216	2	51.32 ± 15.75[a]	148.97 ± 16.49[b]	103.73 ± 29.16[c]
16	Pyroglutamic acid	17.37	59, **156**, 230, 258	2	5.28 ± 0.94[a]	17.40 ± 2.28[b]	33.00 ± 9.31[c]

17	Serine	13.45	100, 133, **204**, 218	3	1305.91 ± 306.68[a]	43.10 ± 5.43[b]	54.72 ± 15.26[b]
18	Threonine	11.75	57, **117**, 130, 219	2	400.02 ± 108.63[a]	29.47 ± 3.49[b]	42.02 ± 12.02[b]
		14.08	101, 117, **218**, 291	3			
19	Tryptophan	30.92	100, **202**, 218, 291	3	ND	2.96 ± 0.59[b]	4.44 ± 2.75[b]
20	Tyrosine	26.37	100, 179, **218**, 280	3	1.03 ± 0.15[a]	22.60 ± 3.00[b]	36.46 ± 14.88[c]
21	Valine	6.72	55, **72**, 130, 146	1	69.38 ± 17.30[a]	50.55 ± 7.69[a]	91.39 ± 24.40[b]
		9.74	100, 133, **144**, 218	2			
	**Organic acid**						
22	Aminomalonic acid	16.16	133, 174, **218**, 320	3	1.23 ± 0.44[a]	4.65 ± 0.67[b]	10.64 ± 3.68[c]
23	Fumaric acid	13.27	133, 155, 217, **245**	2	1.68 ± 0.45[a]	5.33 ± 0.50[b]	3.87 ± 0.80[c]
24	Lactic acid	6.05	**117**, 133, 191, 219	2	106.90 ± 31.72[a]	326.20 ± 59.15[b]	752.36 ± 103.85[c]
25	Malic acid	16.67	133, **233**, 245, 335	3	3.96 ± 1.06[a]	12.59 ± 1.06[b]	10.42 ± 2.19[c]
26	Succinic acid	12.31	55, 129, 172, **247**	2	ND	0.28 ± 0.12[b]	0.54 ± 0.23[c]
	**Purine**						
27	Hypoxanthine	23.74	193, 206, **265**, 280	2	9.16 ± 3.18[a]	277.38 ± 15.69[b]	103.39 ± 29.76[c]
28	Inosine	36.11	**217**, 230, 245, 281	4	2.10 ± 1.64[a]	2.59 ± 0.20[a]	11.70 ± 2.62[b]
29	Guanine	29.58	264, 280, **352**, 367	3	0.34 ± 0.20[a]	0.63 ± 0.06[a]	13.01 ± 4.54[b]
30	Xanthine	27.81	279, 294, **353**, 368	3	ND	14.63 ± 1.28[b]	ND
	**Pyrimidine**						
31	Uracil	12.86	99, 113, **241**, 256	2	0.43 ± 0.14[a]	21.81 ± 2.88[b]	20.43 ± 5.47[b]
32	Uridine	34.33	103, 169, **217**, 259	3	2.44 ± 0.65[a]	39.27 ± 4.34[b]	17.18 ± 4.81[c]
	**Sugar**						
33	Glucose	27.13	129, 191, **204**, 217	5	34.32 ± 3.38[a]	47.94 ± 3.83[b]	51.33 ± 19.24[b]

34	Glyceric acid	12.68	133, **189**, 292, 307	3	0.34 ± 0.17[a]	3.53 ± 0.84[b]	0.95 ± 0.22[c]
35	Glucose-6-phosphate	33.49	204, 299, 357, **387**	6	6.36 ± 2.55[a]	25.50 ± 2.10[b]	40.98 ± 11.97[c]
36	Mannose-6-phosphate	32.48	299, 357, **387**, 471	6(1MEOX)	1.40 ± 0.57[a]	5.35 ± 0.78[b]	5.89 ± 1.29[b]
37	Ribose	21.02	**103**, 189, 217, 307	4(1MEOX)	ND	3.27 ± 0.29[b]	12.05 ± 4.36[c]
	**Other**						
38	Creatinine	18.10	100, **115**, 143, 329	3	3.92 ± 1.07[a]	10.96 ± 1.49[b]	15.52 ± 3.70[c]
39	Phosphoric acid	22.88	103, 299, 315, **357**	4	68.59 ± 12.93[a]	19.67 ± 6.06[b]	15.94 ± 7.56[b]

Supplementary Table S2. Lipid species identified from extracts of human epidermal melanocytes (HEMn-LP) and melanoma cells (A375, A2058) using nanoESI-MS/MS.

Lipid molecular species	Ion species	m/z	MS/MS fragment ion (m/z)
**Positive ion mode**			
Phosphatidylcholine (PC)			
PC (14:0/16:0)	$[M + H]^+$	706	**450** [lyso-PC(14:0) – H_2O + H]$^+$; **468** [lyso-PC(14:0) + H]$^+$; **478** [lyso-PC(16:0) – H_2O + H]$^+$; **496** [lyso-PC(16:0) + H]$^+$; **523** [M – $C_5H_{14}NO_4P$ + H]$^+$; **647** [M – C_3H_9N + H]$^+$; **688** [M – H_2O + H]$^+$
PC (16:0/16:1)	$[M + H]^+$	732	**476** [lyso-PC(16:1) – H_2O + H]$^+$; **478** [lyso-PC(16:0) – H_2O + H]$^+$; **494** [lyso-PC(16:1) + H]$^+$; **496** [lyso-PC(16:0) + H]$^+$; **549** [M – $C_5H_{14}NO_4P$ + H]$^+$; **673** [M – C_3H_9N + H]$^+$; **714** [M – H_2O + H]$^+$
PC (16:0/16:0)	$[M + H]^+$	734	**478** [lyso-PC(16:0) – H_2O + H]$^+$; **496** [lyso-PC(16:0) + H]$^+$; **551** [M – $C_5H_{14}NO_4P$ + H]$^+$; **675** [M – C_3H_9N + H]$^+$; **716** [M – H_2O + H]$^+$
PC (16:1/18:1)	$[M + H]^+$	758	**476** [lyso-PC(16:1) – H_2O + H]$^+$; **494** [lyso-PC(16:1) + H]$^+$; **504** [lyso-PC(18:1) – H_2O + H]$^+$; **522** [lyso-PC(18:1) + H]$^+$; **575** [M – $C_5H_{14}NO_4P$ + H]$^+$; **699** [M – C_3H_9N + H]$^+$; **740** [M – H_2O + H]$^+$
PC (16:0/18:1)	$[M + H]^+$	760	**478** [lyso-PC(16:0) – H_2O + H]$^+$; **496** [lyso-PC(16:0) + H]$^+$; **504** [lyso-PC(18:1) – H_2O + H]$^+$; **522** [lyso-PC(18:1) + H]$^+$; **577** [M – $C_5H_{14}NO_4P$ + H]$^+$; **701** [M – C_3H_9N + H]$^+$; **742** [M – H_2O + H]$^+$
PC (16:0/18:0)	$[M + H]^+$	762	**478** [lyso-PC(16:0) – H_2O + H]$^+$; **496** [lyso-PC(16:0) + H]$^+$; **506** [lyso-PC(18:0) – H_2O + H]$^+$; **524** [lyso-PC(18:0) + H]$^+$; **579** [M – $C_5H_{14}NO_4P$ + H]$^+$; **703** [M – C_3H_9N + H]$^+$; **744** [M – H_2O + H]$^+$
PC (16:0/19:0)	$[M + H]^+$	776	**478** [lyso-PC(16:0) – H_2O + H]$^+$; **496** [lyso-PC(16:0) + H]$^+$; **520** [lyso-PC(19:0) – H_2O + H]$^+$; **538** [lyso-PC(19:0) + H]$^+$; **593** [M – $C_5H_{14}NO_4P$ + H]$^+$; **717** [M – C_3H_9N + H]$^+$; **758** [M – H_2O + H]$^+$
PC (18:1/18:1)	$[M + H]^+$	786	**504** [lyso-PC(18:1) – H_2O + H]$^+$; **522** [lyso-PC(18:1) + H]$^+$; **603** [M – $C_5H_{14}NO_4P$

			+ $H]^+$; **727** [M – C_3H_9N + $H]^+$; **768** [M – H_2O + $H]^+$
PC (18:0/18:1)	$[M + H]^+$	788	**504** [lyso-PC(18:1) – H_2O + $H]^+$; **506** [lyso-PC(18:0) – H_2O + $H]^+$; **522** [lyso-PC(18:1) + $H]^+$; **524** [lyso-PC(18:0) + $H]^+$; **605** [M – $C_5H_{14}NO_4P$ + $H]^+$; **729** [M – C_3H_9N + $H]^+$; **770** [M – H_2O + $H]^+$
Plasmenylphosphatidylcholine (plasmenyl-PC)			
plasmenyl-PC (16:0/18:0)	$[M + Na]^+$	768	**279** [(M + Na) – $C_5H_{14}NO_4P$ – Na – C18:0$]^+$; **563** [(M + Na) – $C_5H_{14}NO_4P$ – Na + $H]^+$; **585** [(M + Na) – $C_5H_{14}NO_4P]^+$; **709** [(M + Na) – $C_3H_9N]^+$
Plasmenylphosphatidylethanolamine (plasmenyl-PE)			
plasmenyl-PE (16:0/22:6)	$[M + H]^+$	748	**266** [C16:0 ether + $C_2H_8NO_3P$ – $H_3PO_4]^+$; **364** [C16:0 ether + $C_2H_8NO_3P]^+$; **385**[lyso(p-16:0) – $C_2H_8NO_3P$ + H$]^+$; **607** [M – H_2O + $H]^+$
plasmenyl-PE (16:0/22:5)	$[M + Na]^+$	772	**386** [C16:0 ether + $C_2H_8NO_3P$ + $Na]^+$; **631** [M – $C_2H_8NO_3P$ + $Na]^+$; **649** [M – $C_2H_8NO_3P$ + H_2O + $Na]^+$; **729** [M – C_2H_5N + $Na]^+$; **754** [M – H_2O + Na$]^+$
Sphingomyelin (SM)			
SM (d18:1/22:0)	$[M + Na]^+$	809	**626** [M – $C_5H_{14}NO_4P]^+$; **732** [M – C_3H_9N – $H_2O]^+$; **750** [M – $C_3H_9N]^+$; **791** [M – $H_2O]^+$
SM (d18:0/22:0)	$[M + Na]^+$	811	**628** [M – $C_5H_{14}NO_4P]^+$; **734** [M – C_3H_9N – $H_2O]^+$; **752** [M – $C_3H_9N]^+$; **793** [M – $H_2O]^+$
**Negative ion mode**			
Ceramide (Cer)			
Cer (d18:1/16:0)	$[M - H]^-$	536	**237** [M – H – $299]^-$; **254** [M – H – $282]^-$; **255** [M – H – $281]^-$; **280** [M – H – $256]^-$; **296** [M – H – $240]^-$; **488** [M – H – CH_2O – $H_2O]^-$; **504** [M – H – $CH_3OH]^-$; **506** [M – H – $CH_2O]^-$; **518** [M – H – $H_2O]^-$
Cer (d18:1/18:1)	$[M - H]^-$	562	**263** [M – H – $299]^-$; **280** [M – H – $282]^-$; **281** [M – H – $281]^-$; **306** [M – H – $256]^-$; **322** [M – H – $240]^-$; **514** [M – H – CH_2O – $H_2O]^-$; **530** [M – H – $CH_3OH]^-$; **532**

			[M – H – CH_2O]$^-$; **544** [M – H – H_2O]$^-$
Cer (d18:1/18:0)	$[M - H]^-$	564	**265** [M – H – 299]$^-$; **282** [M – H – 282]$^-$; **283** [M – H – 281]$^-$; **308** [M – H – 256]$^-$; **324** [M – H – 240]$^-$; **516** [M – H – CH_2O – H_2O]$^-$; **532** [M – H – CH_3OH]$^-$; **534** [M – H – CH_2O]$^-$; **546** [M – H – H_2O]$^-$
Cer (d18:1/20:0)	$[M - H]^-$	592	**293** [M – H – 299]$^-$; **310** [M – H – 282]$^-$; **311** [M – H – 281]$^-$; **336** [M – H – 256]$^-$; **352** [M – H – 240]$^-$; **544** [M – H – CH_2O – H_2O]$^-$; **560** [M – H – CH_3OH]$^-$; **562** [M – H – CH_2O]$^-$; **574** [M – H – H_2O]$^-$
Cer (d18:1/22:0)	$[M - H]^-$	620	**321** [M – H – 299]$^-$; **338** [M – H – 282]$^-$; **339** [M – H – 281]$^-$; **364** [M – H – 256]$^-$; **380** [M – H – 240]$^-$; **572** [M – H – CH_2O – H_2O]$^-$; **588** [M – H – CH_3OH]$^-$; **590** [M – H – CH_2O]$^-$; **602** [M – H – H_2O]$^-$
Plasmenylphosphatidylethanolamine (plasmenyl-PE)			
plasmenyl-PE (16:0/18:1)	$[M - H]^-$	700	**281** [C18:1 – H]$^-$; **418** [lyso-PE(P-16:0) – H_2O – H]$^-$; **436** [lyso-PE(P-16:0) – H]$^-$
plasmenyl-PE (16:0/20:4)	$[M - H]^-$	722	**303** [C20:4 – H]$^-$; **418** [lyso-PE(P-16:0) – H_2O – H]$^-$; **436** [lyso-PE(P-16:0) – H]$^-$
plasmenyl-PE (18:0/20:4)	$[M - H]^-$	750	**303** [C20:4 – H]$^-$; **446** [lyso-PE(P-18:0) – H_2O – H]$^-$; **464** [lyso-PE(P-18:0) – H]$^-$
plasmenyl-PE (18:0/22:5)	$[M - H]^-$	776	**329** [C22:5 – H]$^-$; **446** [lyso-PE(P-18:0) – H_2O – H]$^-$; **464** [lyso-PE(P-18:0) – H]$^-$
Phosphatidylethanolamine (PE)			
PE (16:1/18:1)	$[M - H]^-$	714	**253** [C16:1 – H]$^-$; **281** [C18:1 – H]$^-$; **432** [lyso-PE(16:1) – H_2O – H]$^-$; **450** [lyso-PE(16:1) – H]$^-$; **460** [lyso-PE(18:1) – H_2O – H]$^-$; **478** [lyso-PE(18:1) – H]$^-$
PE (16:0/18:1)	$[M - H]^-$	716	**255** [C16:0 – H]$^-$; **281** [C18:1 – H]$^-$; **434** [lyso-PE(16:0) – H_2O – H]$^-$; **452** [lyso-PE(16:0) – H]$^-$; **460** [lyso-PE(18:1) – H_2O – H]$^-$; **478** [lyso-PE(18:1) – H]$^-$
PE (18:1/18:1)	$[M - H]^-$	742	**281** [C18:1 – H]$^-$; **460** [lyso-PE(18:1) – H_2O – H]$^-$; **478** [lyso-PE(18:1) – H]$^-$
PE (18:0/18:1)	$[M - H]^-$	744	**281** [C18:1 – H]$^-$; **283** [C18:0 – H]$^-$; **460** [lyso-PE(18:1) – H_2O – H]$^-$; **462** [lyso-PE(18:0) – H_2O – H]$^-$; **478** [lyso-PE(18:1) – H]$^-$; **480** [lyso-PE(18:0) – H]$^-$
PE (18:1/20:4)	$[M - H]^-$	764	**281** [C18:1 – H]$^-$; **303** [C20:4 – H]$^-$; **460** [lyso-PE(18:1) – H_2O – H]$^-$; **478** [lyso-PE(18:1) – H]$^-$; **482** [lyso-PE(20:4) – H_2O – H]$^-$; **500** [lyso-PE(20:4) – H]$^-$

PE (18:0/20:4)	[M − H]$^-$	766	**283** [C18:0 – H]$^-$; **303** [C20:4 – H]$^-$; **462** [lyso-PE(18:0) – H_2O – H]$^-$; **480** [lyso-PE(18:0) – H]$^-$; **482** [lyso-PE(20:4) – H_2O – H]$^-$; **500** [lyso-PE(20:4) – H]$^-$
PE (17:0/22:5)	[M − H]$^-$	778	**269** [C17:0 – H]$^-$; **329** [C22:5 – H]$^-$; **448** [lyso-PE(17:0) – H_2O – H]$^-$; **466** [lyso-PE(17:0) – H]$^-$; **508** [lyso-PE(22:5) – H_2O – H]$^-$; **526** [lyso-PE(22:5) – H]$^-$
PE (18:0/22:5)	[M − H]$^-$	792	**283** [C18:0 – H]$^-$; **329** [C22:5 – H]$^-$; **462** [lyso-PE(18:0) – H_2O – H]$^-$; **480** [lyso-PE(18:0) – H]$^-$; **508** [lyso-PE(22:5) – H_2O – H]$^-$; **526** [lyso-PE(22:5) – H]$^-$
Cardiolipin (CL)			
CL (18:1/18:1/18:1/18:1)	[M − 2H]$^{2-}$	727	**281** [C18:1 – H]$^-$; **417** [C18:1 + $C_3H_6PO_4$ – H]$^-$; **463** [lyso-CL(FA1/FA2) – 2H]$^{2-}$ ‖ [lyso-CL(FA3/FA4) – 2H]$^{2-}$; **595** [lyso-CL(FA2/FA3/FA4) – 2H]$^{2-}$ ‖ [lyso-CL(FA1/FA2/FA4) – 2H]$^{2-}$
Phosphatidylglycerol (PG)			
PG (16:1/18:1)	[M − H]$^-$	745	**253** [C16:1 – H]$^-$; **281** [C18:1 – H]$^-$; **389** [lyso-PG(16:1) – $C_3H_6O_2$ – H]$^-$; **417** [lyso-PG(18:1) – $C_3H_6O_2$ – H]$^-$; **463** [lyso-PG(16:1) – H_2O – H]$^-$; **481** [lyso-PG(16:1) – H]$^-$; **491** [lyso-PG(18:1) – H_2O – H]$^-$; **509** [lyso-PG(18:1) – H]$^-$
PG (16:0/18:1)	[M − H]$^-$	747	**255** [C16:0 – H]$^-$; **281** [C18:1 – H]$^-$; **391** [lyso-PG(16:0) – $C_3H_6O_2$ – H]$^-$; **417** [lyso-PG(18:1) – $C_3H_6O_2$ – H]$^-$; **465** [lyso-PG(16:0) – H_2O – H]$^-$; **483** [lyso-PG(16:0) – H]$^-$; **491** [lyso-PG(18:1) – H_2O – H]$^-$; **509** [lyso-PG(18:1) – H]$^-$
PG (18:1/18:2)	[M − H]$^-$	771	**279** [C18:2 – H]$^-$; **281** [C18:1 – H]$^-$; **415** [lyso-PG(18:2) – $C_3H_6O_2$ – H]$^-$; **417** [lyso-PG(18:1) – $C_3H_6O_2$ – H]$^-$; **489** [lyso-PG(18:2) – H_2O – H]$^-$; **491** [lyso-PG(18:1) – H_2O – H]$^-$; **507** [lyso-PG(18:2) – H]$^-$; **509** [lyso-PG(18:1) – H]$^-$
PG (18:1/18:1)	[M − H]$^-$	773	**281** [C18:1 – H]$^-$; **417** [lyso-PG(18:1) – $C_3H_6O_2$ – H]$^-$; **491** [lyso-PG(18:1) – H_2O – H]$^-$; **509** [lyso-PG(18:1) – H]$^-$
PG (18:0/18:1)	[M − H]$^-$	775	**281** [C18:1 – H]$^-$; **283** [C18:0 – H]$^-$; **417** [lyso-PG(18:1) – $C_3H_6O_2$ – H]$^-$; **419** [lyso-PG(18:0) – $C_3H_6O_2$ – H]$^-$; **491** [lyso-PG(18:1) – H_2O – H]$^-$; **493** [lyso-PG(18:0) – H_2O – H]$^-$; **509** [lyso-PG(18:1) – H]$^-$; **511** [lyso-PG(18:0) – H]$^-$
PG (18:1/20:1)	[M − H]$^-$	801	**281** [C18:1 – H]$^-$; **309** [C20:1– H]$^-$; **417** [lyso-PG(18:1) – $C_3H_6O_2$ – H]$^-$; **445** [lyso-PG(20:1) – $C_3H_6O_2$ – H]$^-$; **491** [lyso-PG(18:1) – H_2O – H]$^-$; **509** [lyso-PG(18:1) – H]$^-$; **519** [lyso-PG(20:1) – H_2O – H]$^-$; **537** [lyso-PG(20:1) – H]$^-$

PG (18:0/22:6)	$[M - H]^-$	821	**283** [C18:0 – H]$^-$; **327** [C22:6– H]$^-$; **419** [lyso-PG(18:0) – $C_3H_6O_2$ – H]$^-$; **463** [lyso-PG(22:6) – $C_3H_6O_2$ – H]$^-$; **493** [lyso-PG(18:0) – H_2O – H]$^-$; **511** [lyso-PG(18:0) – H]$^-$; **537** [lyso-PG(22:6) – H_2O – H]$^-$; **555** [lyso-PG(22:6) – H]$^-$
Phosphatidylserine (PS)			
PS (16:1/18:1)	$[M - H]^-$	758	**253** [C16:1 – H]$^-$; **281** [C18:1 – H]$^-$; **389** [lyso-PS(16:1) – $C_3H_5NO_2$ – H]$^-$; **407** [lysoPS(16:1) – $C_3H_5NO_2$ + H_2O – H]$^-$; **417** [lyso-PS(18:1) – $C_3H_5NO_2$ – H]$^-$; **435** [lyso-PS(18:1) – $C_3H_5NO_2$ + H_2O – H]$^-$; **671** [M – $C_3H_5NO_2$ – H]$^-$
PS (16:0/18:1)	$[M - H]^-$	760	**225** [C16:0 – H]$^-$; **281** [C18:1 – H]$^-$; **391** [lyso-PS(16:0) – $C_3H_5NO_2$ – H]$^-$; **409** [lyso-PS(16:0) – $C_3H_5NO_2$ + H_2O – H]$^-$; **417** [lyso-PS(18:1) – $C_3H_5NO_2$ – H]$^-$; **435** [lyso-PS(18:1) – $C_3H_5NO_2$ + H_2O – H]$^-$; **673** [M – $C_3H_5NO_2$ – H]$^-$
PS (16:0/18:0)	$[M - H]^-$	762	**255** [C16:0 – H]$^-$; **283** [C18:0 – H]$^-$; **391** lyso-PS(16:0) – $C_3H_5NO_2$ – H]$^-$; **409** [lyso-PS(16:0) – $C_3H_5NO_2$ + H_2O – H]$^-$; **419** [lyso-PS(18:0) – $C_3H_5NO_2$ – H]$^-$; **437** [lyso-PS(18:0) – $C_3H_5NO_2$ + H_2O – H]$^-$; **675** [M – $C_3H_5NO_2$ – H]$^-$
PS (18:1/18:2)	$[M - H]^-$	784	**279** [C18:2 – H]$^-$; **281** [C18:1 – H]$^-$; **415** [lyso-PS(18:2) – $C_3H_5NO_2$ – H]$^-$; **417** [lyso-PS(18:1) $C_3H_5NO_2$ – H]$^-$; **433** [lyso-PS(18:2) – $C_3H_5NO_2$ + H_2O – H]$^-$; **435** [lyso-PS(18:1) – $C_3H_5NO_2$ + H_2O – H]$^-$; **697** [M – $C_3H_5NO_2$ – H]$^-$
PS (18:1/18:1)	$[M - H]^-$	786	**281** [C18:1 – H]$^-$; **417** [lyso-PS(18:1) – $C_3H_5NO_2$ – H]$^-$; **435** [lyso-PS(18:1) – $C_3H_5NO_2$ + H_2O – H]$^-$; **699** [M – $C_3H_5NO_2$ – H]$^-$
PS (18:0/18:1)	$[M - H]^-$	788	**281** [C18:1 – H]$^-$; **283** [C18:0 – H]$^-$; **417** [lyso-PS(18:1) $C_3H_5NO_2$ – H]$^-$; **419** [lyso-PS(18:0) – $C_3H_5NO_2$ – H]$^-$; **435** [lyso-PS(18:1) – $C_3H_5NO_2$ + H_2O – H]$^-$; **437** [lyso-PS(18:0)) – $C_3H_5NO_2$ + H_2O – H]$^-$; **701** [M – $C_3H_5NO_2$ – H]$^-$
PS (18:0/18:0)	$[M - H]^-$	790	**283** [C18:0 – H]$^-$; **419** [lyso-PS(18:0) – $C_3H_5NO_2$ – H]$^-$; **437** [lyso-PS(18:0) – $C_3H_5NO_2$ + H_2O – H]$^-$; **703** [M – $C_3H_5NO_2$ – H]$^-$
PS (18:1/20:4)	$[M - H]^-$	808	**281** [C18:1 – H]$^-$; **303** [C20:4 – H]$^-$; **417** [lyso-PS(18:1) – $C_3H_5NO_2$ – H]$^-$; **435** [lyso-PS(18:1) – $C_3H_5NO_2$ + H_2O – H]$^-$; **439** [lyso-PS(20:4) – $C_3H_5NO_2$ – H]$^-$; **457** [lyso-PS(20:4) – $C_3H_5NO_2$ + H_2O – H]$^-$; **721** [M – $C_3H_5NO_2$ – H]$^-$
PS (18:0/20:4)	$[M - H]^-$	810	**283** [C18:0 – H]$^-$; **303** [C20:4 – H]$^-$; **419** [lyso-PS(18:0) – $C_3H_5NO_2$ – H]$^-$; **437** [lyso-PS(18:0) – $C_3H_5NO_2$ + H_2O – H]$^-$; **439** [lyso-PS(20:4) – $C_3H_5NO_2$ – H]$^-$; **457** [lyso-PS(20:4) – $C_3H_5NO_2$ + H_2O – H]$^-$; **723** [M – $C_3H_5NO_2$ – H]$^-$
PS (18:0/20:3)	$[M - H]^-$	812	**283** [C18:0 – H]$^-$; **305** [C20:3 – H]$^-$; **419** [lyso-PS(18:0) – $C_3H_5NO_2$ – H]$^-$; **437** [lyso-PS(18:0) – $C_3H_5NO_2$ + H_2O – H]$^-$; **441** [lyso-PS(20:3) – $C_3H_5NO_2$ – H]$^-$; **459**

			[lyso-PS(20:3) – $C_3H_5NO_2$ + H_2O – H]$^-$; **725** [M – $C_3H_5NO_2$ – H]$^-$
PS (18:1/20:1)	[M − H]$^-$	814	**281** [C18:1 – H]$^-$; **309** [C20:1 – H]$^-$; **417** [lyso-PS(18:1) – $C_3H_5NO_2$ – H]$^-$; **435** [lyso-PS(18:1) – $C_3H_5NO_2$ + H_2O – H]$^-$; **445** [lyso-PS(20:1) – $C_3H_5NO_2$ – H]$^-$; **463** [lyso-PS(20:1) – $C_3H_5NO_2$ + H_2O – H]$^-$; **727** [M – $C_3H_5NO_2$ – H]$^-$
PS (18:1/20:0)	[M − H]$^-$	816	**281** [C18:1 – H]$^-$; **311** [C20:0 – H]$^-$; **417** [lyso-PS(18:1) – $C_3H_5NO_2$ – H]$^-$; **435** [lyso-PS(18:1) – $C_3H_5NO_2$ + H_2O – H]$^-$; **447** [lyso-PS(20:0) – $C_3H_5NO_2$ – H]$^-$; **465** [lyso-PS(20:0) – $C_3H_5NO_2$ + H_2O – H]$^-$; **729** [M – $C_3H_5NO_2$ – H]$^-$
PS (18:1/22:1)	[M − H]$^-$	842	**281** [C18:1 – H]$^-$; **337** [C22:1 – H]$^-$; **417** [lyso-PS(18:1) – $C_3H_5NO_2$ – H]$^-$; **435** [lyso-PS(18:1) – $C_3H_5NO_2$ + H_2O – H]$^-$; **473** [lyso-PS(22:1) – $C_3H_5NO_2$ – H]$^-$; **491** [lyso-PS(22:1) – $C_3H_5NO_2$ + H_2O – H]$^-$; **755** [M – $C_3H_5NO_2$ – H]$^-$
PS (18:1/22:0)	[M − H]$^-$	844	**281** [C18:1 – H]$^-$; **339** [C22:0 – H]$^-$; **417** [lyso-PS(18:1) – $C_3H_5NO_2$ – H]$^-$; **435** [lyso-PS(18:1) – $C_3H_5NO_2$ + H_2O – H]$^-$; **475** [lyso-PS(22:0) – $C_3H_5NO_2$ – H]$^-$; **493** [lyso-PS(22:0) – $C_3H_5NO_2$ + H_2O – H]$^-$; **757** [M – $C_3H_5NO_2$ – H]$^-$
PS (18:1/24:1)	[M − H]$^-$	870	**281** [C18:1 – H]$^-$; **365** [C24:1 – H]$^-$; **417** [lyso-PS(18:1) – $C_3H_5NO_2$ – H]$^-$; **435** [lyso-PS(18:1) – $C_3H_5NO_2$ + H_2O – H]$^-$; **501** [lyso-PS(24:1) – $C_3H_5NO_2$ – H]$^-$; **519** [lyso-PS(24:1) – $C_3H_5NO_2$ + H_2O – H]$^-$; **783** [M – $C_3H_5NO_2$ – H]$^-$
Phosphatidylinositol (PI)			
PI (16:1/18:1)	[M − H]$^-$	833	**241** [$C_6H_{10}O_8P$]$^-$; **253** [C16:1 – H]$^-$; **281** [C18:1 – H]$^-$; **297** [$C_9H_{14}O_9P$]$^-$; **315** [$C_9H_{16}O_{10}P$]$^-$; **389** [lyso-PI(16:1) – $C_6H_{12}O_6$ – H]$^-$; **417** [lyso-PI(18:1) – $C_6H_{12}O_6$ – H]$^-$; **551** [lyso-PI(16:1) – H_2O – H]$^-$; **569** [lyso-PI(16:1) – H]$^-$; **579** [lyso-PI(18:1) – H_2O – H]$^-$; **597** [lyso-PI(18:1) – H]$^-$
PI (16:0/18:1)	[M − H]$^-$	835	**241** [$C_6H_{10}O_8P$]$^-$; **255** [C16:0 – H]$^-$; **281** [C18:1 – H]$^-$; **297** [$C_9H_{14}O_9P$]$^-$; **315** [$C_9H_{16}O_{10}P$]$^-$; **391** [lyso-PI(16:0) – $C_6H_{12}O_6$ – H]$^-$; **417** [lyso-PI(18:1) – $C_6H_{12}O_6$ – H]$^-$; **553** [lyso-PI(16:0) – H_2O – H]$^-$; **571** [lyso-PI(16:0) – H]$^-$; **579** [lyso-PI(18:1) – H_2O – H]$^-$; **597** [lyso-PI(18:1) – H]$^-$
PI (16:0/18:0)	[M − H]$^-$	837	**241** [$C_6H_{10}O_8P$]$^-$; **255** [C16:0 – H]$^-$; **283** [C18:0 – H]$^-$; **297** [$C_9H_{14}O_9P$]$^-$; **315** [$C_9H_{16}O_{10}P$]$^-$; **391** [lyso-PI(16:0) – $C_6H_{12}O_6$ – H]$^-$; **419** [lyso-PI(18:0) – $C_6H_{12}O_6$ – H]$^-$; **553** [lyso-PI(16:0) – H_2O – H]$^-$; **571** [lyso-PI(16:0) – H]$^-$; **581** [lyso-PI(18:0) – H_2O – H]$^-$; **599** [lyso-PI(18:0) – H]$^-$
PI (18:1/18:2)	[M − H]$^-$	859	**241** [$C_6H_{10}O_8P$]$^-$; **279** [C18:2 – H]$^-$; **281** [C18:1 – H]$^-$; **297** [$C_9H_{14}O_9P$]$^-$; **315** [$C_9H_{16}O_{10}P$]$^-$; **415** [lyso-PI(18:2) – $C_6H_{12}O_6$ – H]$^-$; **417** [lyso-PI(18:1) – $C_6H_{12}O_6$ –

			H]$^-$; **577** [lyso-PI(18:2) – H_2O – H]$^-$; **579** [lyso-PI(18:1) – H_2O – H]$^-$; **595** [lyso-PI(18:2) – H]$^-$; **597** [lyso-PI(18:1) – H]$^-$
PI (18:1/18:1)	[M − H]$^-$	861	**241** [$C_6H_{10}O_8P$]$^-$; **281** [C18:1 – H]$^-$; **297** [$C_9H_{14}O_9P$]$^-$; **315** [$C_9H_{16}O_{10}P$]$^-$; **417** [lyso-PI(18:1) – $C_6H_{12}O_6$ – H]$^-$; **579** [lyso-PI(18:1) – H_2O – H]$^-$; **597** [lyso-PI(18:1) – H]$^-$
PI (18:0/18:1)	[M − H]$^-$	863	**241** [$C_6H_{10}O_8P$]$^-$; **281** [C18:1 – H]$^-$; **283** [C18:0 – H]$^-$; **297** [$C_9H_{14}O_9P$]$^-$; **315** [$C_9H_{16}O_{10}P$]$^-$; **417** [lyso-PI(18:1) – $C_6H_{12}O_6$ – H]$^-$; **419** [lyso-PI(18:0) – $C_6H_{12}O_6$ – H]$^-$; **579** [lyso-PI(18:1) – H_2O – H]$^-$; **581** [lyso-PI(18:0) – H_2O – H]$^-$; **597** [lyso-PI(18:1) – H]$^-$; **599** [lyso-PI(18:0) – H]$^-$
PI (18:0/18:0)	[M − H]$^-$	865	**241** [$C_6H_{10}O_8P$]$^-$; **283** [C18:0 – H]$^-$; **297** [$C_9H_{14}O_9P$]$^-$; **315** [$C_9H_{16}O_{10}P$]$^-$; **419** [lyso-PI(18:0) – $C_6H_{12}O_6$ – H]$^-$; **581** [lyso-PI(18:0) – H_2O – H]$^-$; **599** [lyso-PI(18:0) – H]$^-$
PI (18:1/20:4)	[M − H]$^-$	883	**241** [$C_6H_{10}O_8P$]$^-$; **281** [C18:1 – H]$^-$; **297** [$C_9H_{14}O_9P$]$^-$; **303** [C20:4 – H]$^-$; **315** [$C_9H_{16}O_{10}P$]$^-$; **417** [lyso-PI(18:1) – $C_6H_{12}O_6$ – H]$^-$; **439** [lyso-PI(20:4) – $C_6H_{12}O_6$ – H]$^-$; **579** [lyso-PI(18:1) – H_2O – H]$^-$; **597** [lyso-PI(18:1) – H]$^-$; **601** [lyso-PI(20:4) – H_2O – H]$^-$; **619** [lyso-PI(20:4) – H]$^-$
PI (18:0/20:4)	[M − H]$^-$	885	**241** [$C_6H_{10}O_8P$]$^-$; **283** [C18:0 – H]$^-$; **297** [$C_9H_{14}O_9P$]$^-$; **303** [C20:4 – H]$^-$; **315** [$C_9H_{16}O_{10}P$]$^-$; **419** [lyso-PI(18:0) – $C_6H_{12}O_6$ – H]$^-$; **439** [lyso-PI(20:4) – $C_6H_{12}O_6$ – H]$^-$; **581** [lyso-PI(18:0) – H_2O – H]$^-$; **599** [lyso-PI(18:0) – H]$^-$; **601** [lyso-PI(20:4) – H_2O – H]$^-$; **619** [lyso-PI(20:4) – H]$^-$
PI (18:0/20:3)	[M − H]$^-$	887	**241** [$C_6H_{10}O_8P$]$^-$; **283** [C18:0 – H]$^-$; **297** [$C_9H_{14}O_9P$]$^-$; **305** [C20:3 – H]$^-$; **315** [$C_9H_{16}O_{10}P$]$^-$; **419** [lyso-PI(18:0) – $C_6H_{12}O_6$ – H]$^-$; **441** [lyso-PI(20:3) – $C_6H_{12}O_6$ – H]$^-$; **581** [lyso-PI(18:0) – H_2O – H]$^-$; **599** [lyso-PI(18:0) – H]$^-$; **603** [lyso-PI(20:3) – H_2O – H]$^-$; **621** [lyso-PI(20:3) – H]$^-$
PI (18:0/20:2)	[M − H]$^-$	889	**241** [$C_6H_{10}O_8P$]$^-$; **283** [C18:0 – H]$^-$; **297** [$C_9H_{14}O_9P$]$^-$; **307** [C20:2 – H]$^-$; **315** [$C_9H_{16}O_{10}P$]$^-$; **419** [lyso-PI(18:0) – $C_6H_{12}O_6$ – H]$^-$; **443** [lyso-PI(20:2) – $C_6H_{12}O_6$ – H]$^-$; **581** [lyso-PI(18:0) – H_2O – H]$^-$; **599** [lyso-PI(18:0) – H]$^-$; **605** [lyso-PI(20:2) – H_2O – H]$^-$; **623** [lyso-PI(20:2) – H]$^-$

Supplementary Table S3. Normalized ion intensities of the identified lipids in human epidermal melanocytes (HEMn-LP) and melanoma cell lines (A375, A2058) using DI-MS. Each value represents the mean ± standard deviation (SD) (n = 10). For simplicity, ‘×10e4’ and ‘×10e5’ was omitted from the values for lipids identified in positive mode and negative modes, respectively.

No.	Lipid species	Ion species	m/z	HEMn-LP	A375	A2058
	**Positive ion mode**					
	Phosphatidylcholine (PC)					
1	PC (14:0/16:0)	$[M + H]^+$	706	7.60 ± 2.55[a]	2.45 ± 0.21[b]	5.04 ± 0.68[c]
2	PC (16:0/16:1)	$[M + H]^+$	732	19.20 ± 6.45[a]	8.98 ± 0.61[b]	21.12 ± 3.07[a]
3	PC (16:0/16:0)	$[M + H]^+$	734	14.85 ± 5.25[a]	3.95 ± 0.28[b]	8.58 ± 1.19[c]
4	PC (16:1/18:1)	$[M + H]^+$	758	25.96 ± 8.46[a]	7.05 ± 0.46[b]	18.09 ± 2.75[c]
5	PC (16:0/18:1)	$[M + H]^+$	760	64.05 ± 23.62[a]	25.38 ± 1.78[b]	52.23 ± 7.57[a]
6	PC (16:0/18:0)	$[M + H]^+$	762	15.92 ± 4.64[a]	4.56 ± 0.30[b]	8.38 ± 1.01[c]
7	PC (16:0/19:0)	$[M + H]^+$	776	3.83 ± 1.21[a]	0.68 ± 0.03[b]	1.12 ± 0.18[b]
8	PC (18:1/18:1)	$[M + H]^+$	786	172.49 ± 48.97[a]	26.18 ± 2.10[b]	55.60 ± 7.39[b]
9	PC (18:0/18:1)	$[M + H]^+$	788	113.71 ± 32.12[a]	16.07 ± 1.34[b]	32.52 ± 4.67[b]
	Plasmenylphosphatidylcholine (plasmenyl-PC)					
10	plasmenyl-PC (16:0/18:0)	$[M + Na]^+$	768	4.26 ± 1.36[a]	2.31 ± 0.09[b]	3.90 ± 0.86[a]
	Plasmenylphosphatidylethanolamine (plasmenyl-PE)					
11	plasmenyl-PE (16:0/22:6)	$[M + H]^+$	748	1.72 ± 0.65[a]	1.74 ± 0.11[a]	3.12 ± 0.36[b]
12	plasmenyl-PE (16:0/22:5)	$[M + Na]^+$	772	3.33 ± 1.54[a]	1.86 ± 0.15[b]	5.59 ± 0.86[c]
	Sphingomyelin (SM)					
13	SM (d18:1/22:0)	$[M + Na]^+$	809	10.62 ± 4.33[a]	2.21 ± 0.18[b]	4.79 ± 1.26[b]

14	SM (d18:0/22:0)	$[M + Na]^+$	811	6.05 ± 1.94[a]	0.68 ± 0.04[b]	0.91 ± 0.14[b]
	**Negative ion mode**					
	Ceramide (Cer)					
15	Cer (d18:1/16:0)	$[M - H]^-$	536	46.62 ± 11.13[a]	187.48 ± 34.27[b]	66.73 ± 27.90[a]
16	Cer (d18:1/18:1)	$[M - H]^-$	562	89.30 ± 24.71[a]	72.53 ± 14.24[ab]	55.09 ± 18.35[b]
17	Cer (d18:1/18:0)	$[M - H]^-$	564	73.29 ± 18.12[a]	169.20 ± 31.00[b]	63.56 ± 18.43[a]
18	Cer (d18:1/20:0)	$[M - H]^-$	592	5.37 ± 1.58[a]	8.58 ± 1.64[b]	5.67 ± 1.08[a]
19	Cer (d18:1/22:0)	$[M - H]^-$	620	2.63 ± 0.85[a]	4.84 ± 0.85[b]	2.70 ± 0.69[a]
	Plasmenylphosphatidylethanolamine (plasmenyl-PE)					
20	plasmenyl-PE (16:0/18:1)	$[M - H]^-$	700	29.22 ± 5.12[a]	18.76 ± 1.06[b]	31.35 ± 8.31[a]
21	plasmenyl-PE (16:0/20:4)	$[M - H]^-$	722	33.17 ± 5.87[a]	23.47 ± 1.79[b]	25.29 ± 6.14[b]
22	plasmenyl-PE (18:0/20:4)	$[M - H]^-$	750	38.54 ± 6.58[a]	20.36 ± 1.38[b]	22.64 ± 5.76[b]
23	plasmenyl-PE (18:0/22:5)	$[M - H]^-$	776	22.20 ±6.27[a]	14.04 ± 1.53[b]	29.18 ± 8.76[c]
	Phosphatidylethanolamine (PE)					
24	PE (16:1/18:1)	$[M - H]^-$	714	11.85 ± 2.77[a]	10.08 ± 0.80[a]	20.00 ± 5.64[b]
25	PE (16:0/18:1)	$[M - H]^-$	716	21.28 ± 3.87[ab]	20.46 ± 1.44[a]	26.05 ± 7.64[b]
26	PE (18:1/18:1)	$[M - H]^-$	742	84.67 ± 16.34[a]	32.35 ± 2.12[b]	63.00 ± 19.25[c]
27	PE (18:0/18:1)	$[M - H]^-$	744	46.23 ± 10.09[a]	32.21 ± 2.17[b]	44.46 ± 12.51[a]
28	PE (18:1/20:4)	$[M - H]^-$	764	25.28 ± 3.84[a]	14.96 ± 1.28[b]	24.47 ± 7.09[a]
29	PE (18:0/20:4)	$[M - H]^-$	766	16.79 ± 2.49[a]	17.90 ± 1.02[a]	16.27 ± 4.95[a]
30	PE (17:0/22:5)	$[M - H]^-$	778	13.56 ± 2.66[a]	10.58 ± 0.85[a]	19.31 ± 6.22[b]
31	PE (18:0/22:5)	$[M - H]^-$	792	27.79 ± 4.56[a]	22.57 ± 1.80[a]	36.34 ± 10.76[b]
	Cardiolipin (CL)					

32	CL (18:1/18:1/18:1/18:1)	$[M-2H]^{2-}$	727	23.56 ± 4.09[a]	4.92 ± 0.33[b]	12.44 ± 3.44[c]
	Phosphatidylglycerol (PG)					
33	PG (16:1/18:1)	$[M-H]^-$	745	21.00 ± 4.42[a]	26.33 ± 2.96[a]	55.81 ± 13.95[b]
34	PG (16:0/18:1)	$[M-H]^-$	747	20.30 ± 3.89[a]	49.68 ± 6.88[b]	76.11 ± 16.82[c]
35	PG (18:1/18:2)	$[M-H]^-$	771	10.81 ± 2.20[a]	8.31 ± 1.29[a]	18.14 ± 4.86[b]
36	PG (18:1/18:1)	$[M-H]^-$	773	89.05 ± 15.80[a]	36.78 ± 5.66[b]	136.90 ± 36.70[c]
37	PG (18:0/18:1)	$[M-H]^-$	775	26.71 ± 4.53[a]	25.12 ± 3.06[a]	38.11 ± 9.02[b]
38	PG (18:1/20:1)	$[M-H]^-$	801	9.60 ± 2.89[a]	3.90 ± 0.75[b]	10.35 ± 2.41[a]
39	PG (18:0/22:6)	$[M-H]^-$	821	19.95 ± 3.22[a]	9.12 ± 0.83[b]	20.07 ± 4.31[a]
	Phosphatidylserine (PS)					
40	PS (16:1/18:1)	$[M-H]^-$	758	48.32 ± 9.45[a]	29.13 ± 4.65[b]	49.88 ± 12.17[a]
41	PS (16:0/18:1)	$[M-H]^-$	760	73.91 ± 13.53[a]	92.10 ± 12.54[a]	130.21 ± 23.80[b]
42	PS (16:0/18:0)	$[M-H]^-$	762	28.09 ± 4.47[a]	19.48 ± 2.20[b]	30.21 ± 5.74[a]
43	PS (18:1/18:2)	$[M-H]^-$	784	26.13 ± 6.04[a]	18.16 ± 3.41[b]	17.22 ± 4.42[b]
44	PS (18:1/18:1)	$[M-H]^-$	786	240.79 ± 40.62[a]	115.99 ± 18.54[b]	156.37 ± 36.01[c]
45	PS (18:0/18:1)	$[M-H]^-$	788	487.58 ± 69.41[a]	154.23 ± 13.02[b]	230.89 ± 41.24[c]
46	PS (18:0/18:0)	$[M-H]^-$	790	93.60 ± 15.11[a]	52.11 ± 4.46[b]	85.32 ± 22.88[a]
47	PS (18:1/20:4)	$[M-H]^-$	808	8.63 ± 2.61[a]	7.02 ± 1.30[a]	12.73 ± 3.19[b]
48	PS (18:0/20:4)	$[M-H]^-$	810	13.85 ± 2.08[a]	4.09 ± 0.39[b]	4.37 ± 0.87[b]
49	PS (18:0/20:3)	$[M-H]^-$	812	21.55 ± 2.71[a]	5.46 ± 0.38[b]	4.91 ± 1.26[b]
50	PS (18:1/20:1)	$[M-H]^-$	814	51.79 ± 7.40[a]	6.77 ± 0.66[b]	16.20 ± 4.07[c]
51	PS (18:1/20:0)	$[M-H]^-$	816	24.40 ± 4.33[a]	21.77 ± 2.06[a]	42.63 ± 12.27[b]
52	PS (18:1/22:1)	$[M-H]^-$	842	37.11 ± 5.59[a]	7.48 ± 0.67[b]	14.39 ± 2.94[c]
53	PS (18:1/22:0)	$[M-H]^-$	844	52.20 ± 10.07[a]	36.41 ± 4.10[b]	62.17 ± 18.72[a]
54	PS (18:1/24:1)	$[M-H]^-$	870	23.28 ± 4.81[a]	4.52 ± 0.35[b]	9.80 ± 1.66[c]

	Phosphatidylinositol (PI)					
55	PI (16:1/18:1)	$[M-H]^-$	833	26.95 ± 4.18[a]	18.12 ± 2.06[b]	54.82 ± 11.17[c]
56	PI (16:0/18:1)	$[M-H]^-$	835	23.90 ± 4.09[a]	48.38 ± 4.23[b]	122.77 ± 21.75[c]
57	PI (16:0/18:0)	$[M-H]^-$	837	5.54 ± 1.05[a]	14.62 ± 1.18[b]	24.48 ± 3.56[c]
58	PI (18:1/18:2)	$[M-H]^-$	859	12.69 ± 1.89[a]	11.22 ± 1.14[a]	16.33 ± 3.61[b]
59	PI (18:1/18:1)	$[M-H]^-$	861	88.25 ± 15.73[a]	86.92 ± 8.28[a]	178.04 ± 35.89[b]
60	PI (18:0/18:1)	$[M-H]^-$	863	42.62 ± 7.69[a]	111.70 ± 9.50[b]	139.72 ± 25.15[c]
61	PI (18:0/18:0)	$[M-H]^-$	865	15.87 ± 3.15[a]	54.07 ± 4.56[b]	65.71 ± 11.60[c]
62	PI (18:1/20:4)	$[M-H]^-$	883	48.38 ± 8.30[a]	28.56 ± 3.09[b]	46.99 ± 8.53[a]
63	PI (18:0/20:4)	$[M-H]^-$	885	183.29 ± 32.05[a]	102.89 ± 9.41[b]	62.21 ± 10.45[c]
64	PI (18:0/20:3)	$[M-H]^-$	887	188.29 ± 33.37[a]	51.51 ± 4.67[b]	33.92 ± 5.98[b]
65	PI (18:0/20:2)	$[M-H]^-$	889	39.43 ± 6.99[a]	26.92 ± 2.38[b]	33.52 ± 6.87[a]

Supplementary Table S4. Changes in the relative levels of metabolites in fresh and spent media. The *p*-values were obtained by *t*-test. Each fresh and spent medium sample was taken after 2 days of melanoma cell culture. *, significant difference ($p < 0.05$); **, significant difference ($p < 0.01$); ***, significant difference ($p < 0.001$); ↑, increase in metabolites in spent medium compared to those in fresh medium; ↓, decrease in metabolites in spent medium compared to those in fresh medium. RT, retention time; TMS, trimethylsilylation.

Compound	RT (min)	Mass fragment (m/z)	TMS	A375	A2058
**Alcohol**					
Myo-inositol***	36.33	191, **217**, 305, 318	6	23.27 ± 1.83 (↓)	7.47 ± 2.52 (↓)
**Amino acid**					
Alanine**	8.57	45, **116**, 190, 218	2	25.29 ± 3.77 (↑)	44.35 ± 16.60 (↑)
Glutamic acid***	24.57	218, **246**, 348, 363	3	22.96 ± 3.74 (↑)	37.14 ± 6.12 (↑)
Glutamine***	28.74	**156**, 245, 347, 362	3	32.37 ± 0.91 (↓)	44.05 ± 4.32 (↓)
Isoleucine	14.53	45, **158**, 218, 260	2	35.78 ± 5.90 (↓)	38.19 ± 14.59 (↓)
Leucine	10.2	44, 75, **86**, 188	1	31.17 ± 5.70 (↓)	40.06 ± 23.95 (↓)
	13.84	**158**, 218, 232, 260	2		
Ornithine***	29.8	**142**, 174, 200, 258	4	1.30 ± 0.29 (↑)	2.55 ± 0.56 (↑)
Proline***	14.62	100, 133, **142**, 216	2	79.45 ± 15.91 (↑)	47.29 ± 15.86 (↑)
Pyroglutamic acid**	21.55	45, **156**, 230, 258	2	84.49 ± 18.62 (↑)	119.52 ± 27.79 (↑)
Serine**	16.71	100, **204**, 218, 306	3	29.06 ± 0.87 (↓)	23.52 ± 3.77 (↓)
Tyrosine***	32.86	**218**, 280, 354, 382	3	21.74 ± 13.41 (↑)	52.56 ± 21.49 (↑)
Valine***	8.19	55, **72**, 146, 174	1	24.13 ± 4.70 (↑)	54.45 ± 13.90 (↑)
	12.02	45, 100, **144**, 218	2		
**Organic acid**					

Lactic acid***	7.41	**117**, 133, 191, 219	2	2437.85 ± 207.04 (↑)	3074.88 ± 333.52 (↑)
Malic acid	20.72	133, **233**, 245, 335	3	0.26 ± 0.05 (↑)	0.30 ± 0.16 (↑)
Pyruvic acid**	7.11	89, 100, 115, **174**	1(1MEOX)	18.59 ± 0.38 (↓)	11.51 ± 5.13 (↓)
Succinic acid	15.27	45, 129, 172, **247**	2	0.35 ± 0.17 (↑)	0.44 ± 0.22 (↑)
**Sugar**					
Glucose**	34.01	129, 191, **204**, 217	5	3019.56 ± 333.00 (↓)	2206.99 ± 864.69 (↓)
	31.83	160, 205, 217, **319**	5(1MEOX)		
**Fatty acid**					
Palmitic acid	35.54	117, 132, **313**, 328	1	1.72 ± 0.21 (↓)	1.98 ± 0.48 (↓)

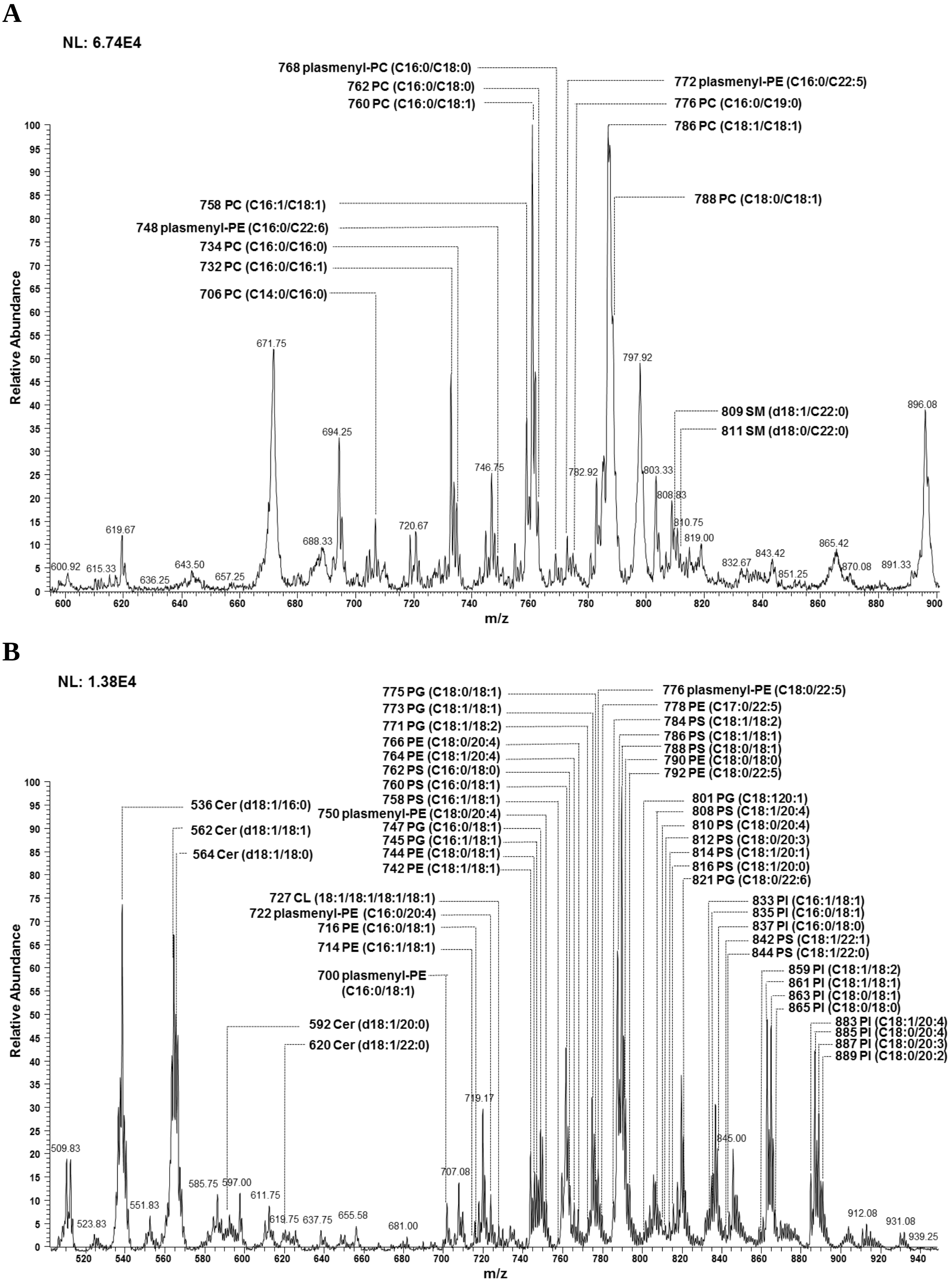

Supplementary Figure S1. Representative spectra of lipid profiles from melanocytes and melanoma cell lines from nanoESI-MS in (A) positive ion mode and (B) negative ion mode

Compound #1

Aminomalonic acid

Retention time (min) : 16.16
Fragmentation ion (m/z) : 133, 174, **218**, 320
Formula : $C_3H_5NO_4$

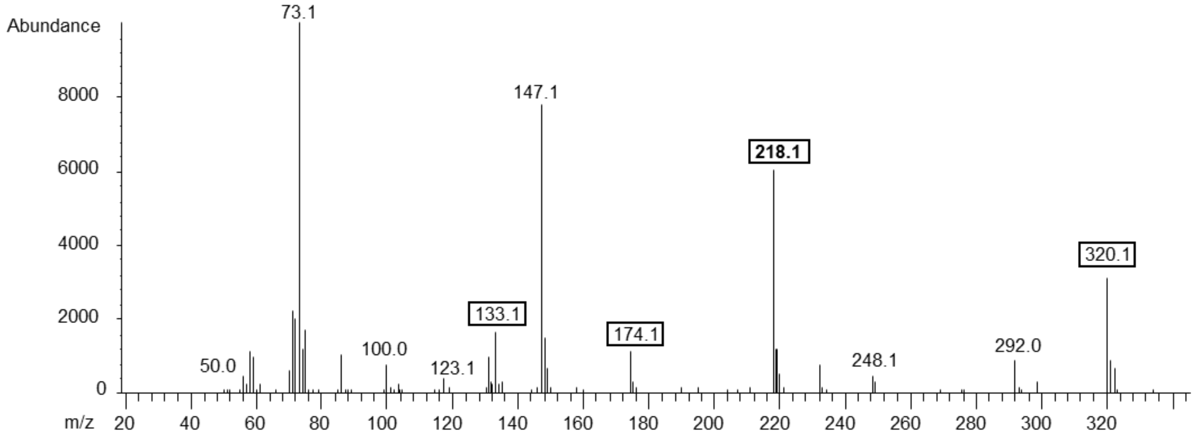

Sample : Aminomalonic acid (3TMS)

Compound #2

[PI(16:0/18:1) - H]$^-$, *m/z* 835

• $C_6H_{12}O_6$: inositol group

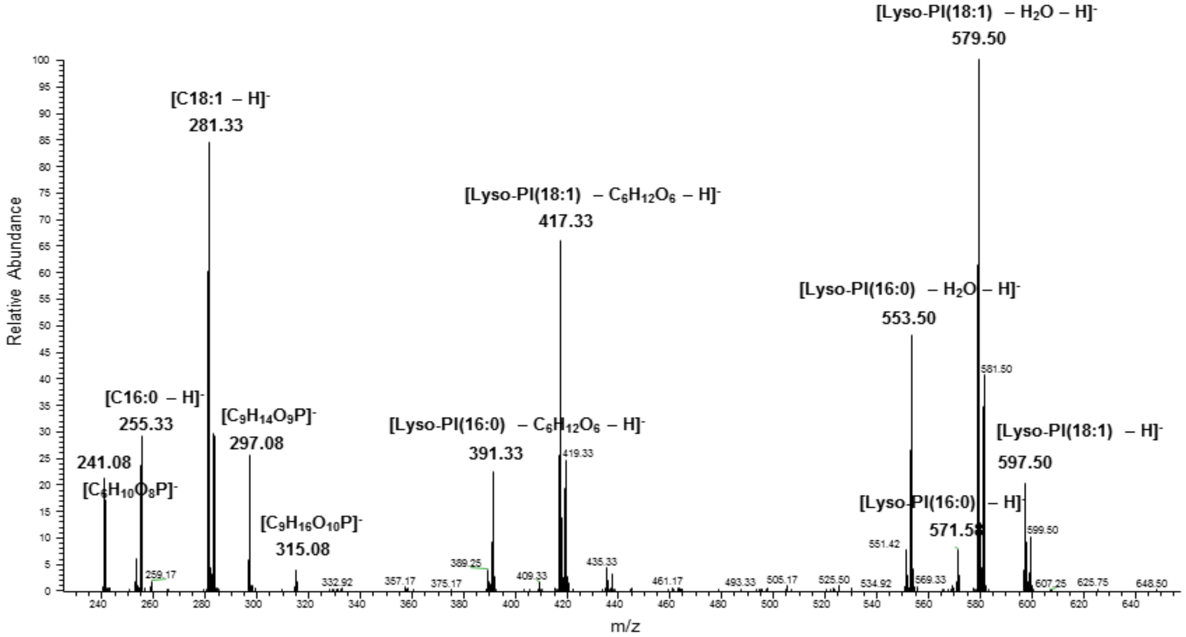

Compound #3

[PI(16:0/18:0) - H]$^-$, *m/z* 837

• $C_6H_{12}O_6$: inositol group

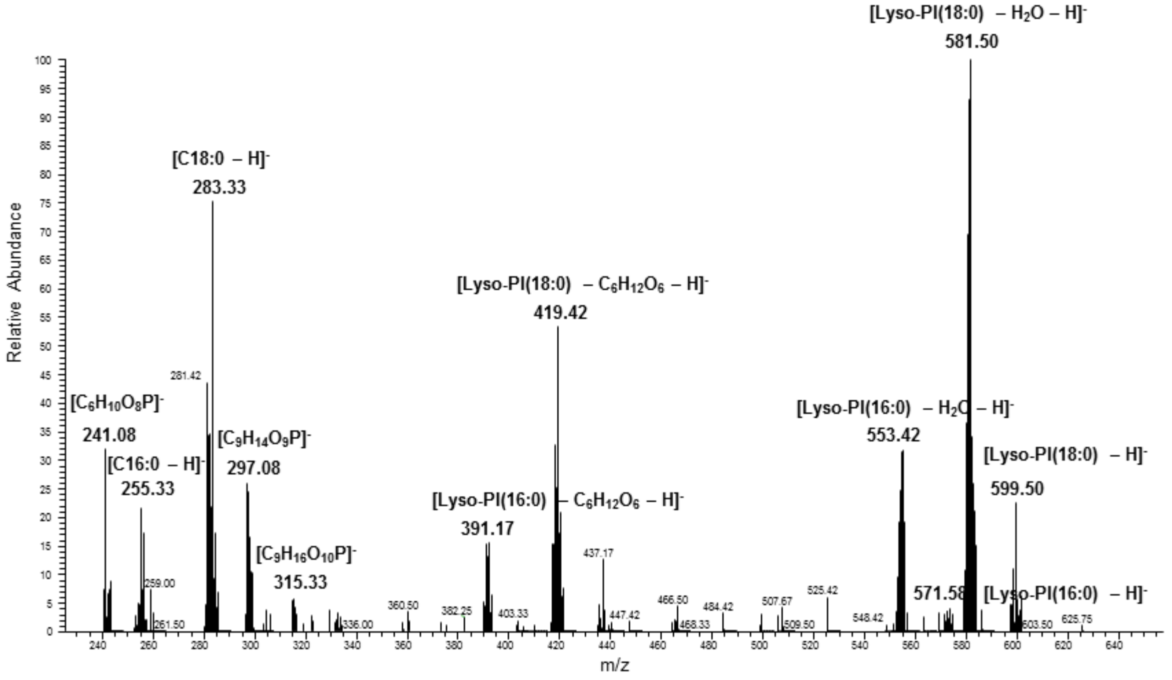

Compound #4

[PI(18:0/18:1) - H]$^-$, *m/z* 863

• $C_6H_{12}O_6$: inositol group

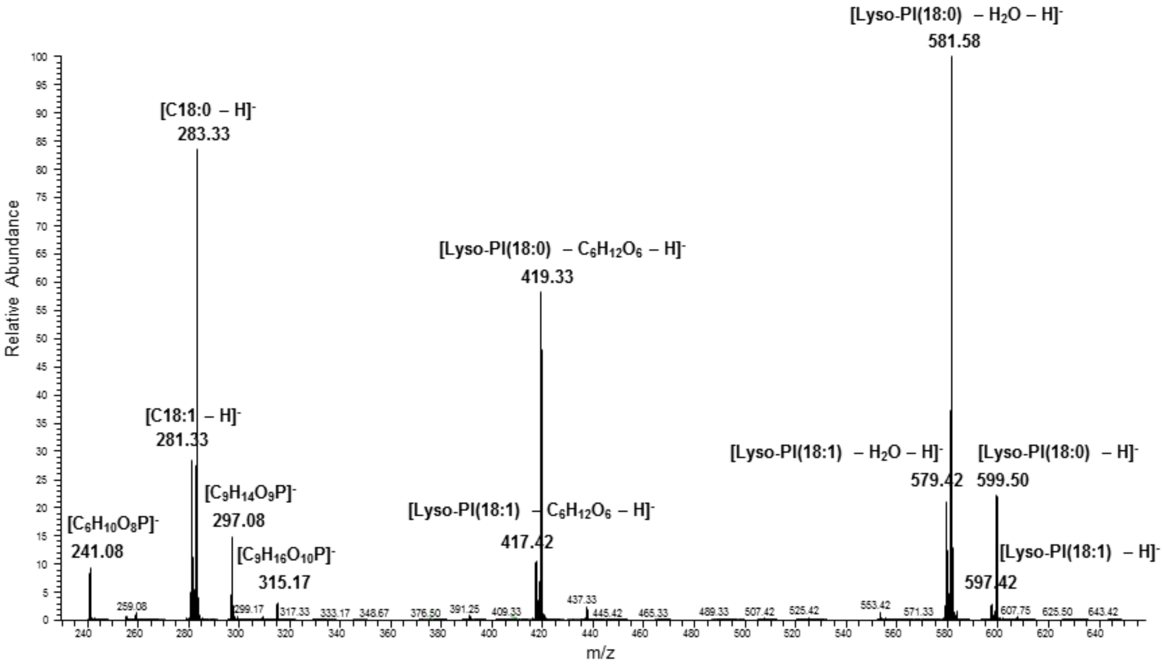

Compound #5

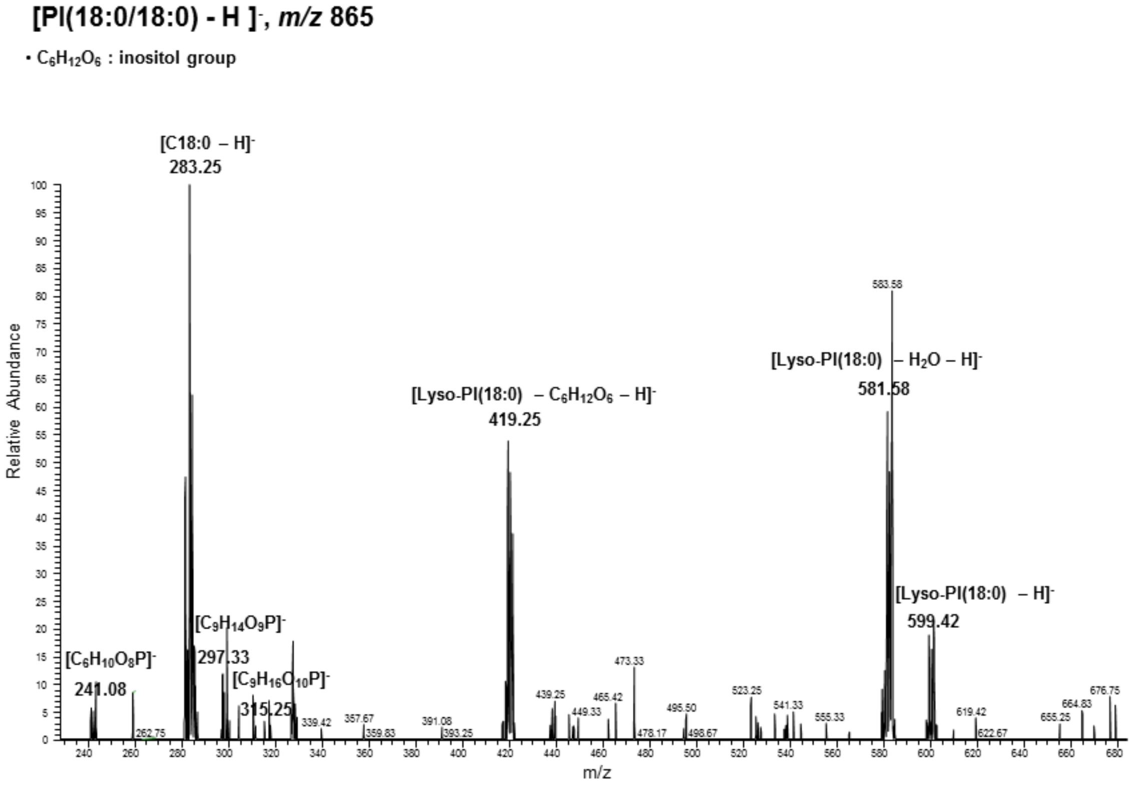

Supplementary Figure S2. Fragmentation mass spectra of the novel metabolite and lipid biomarkers of melanoma with different metastatic potential

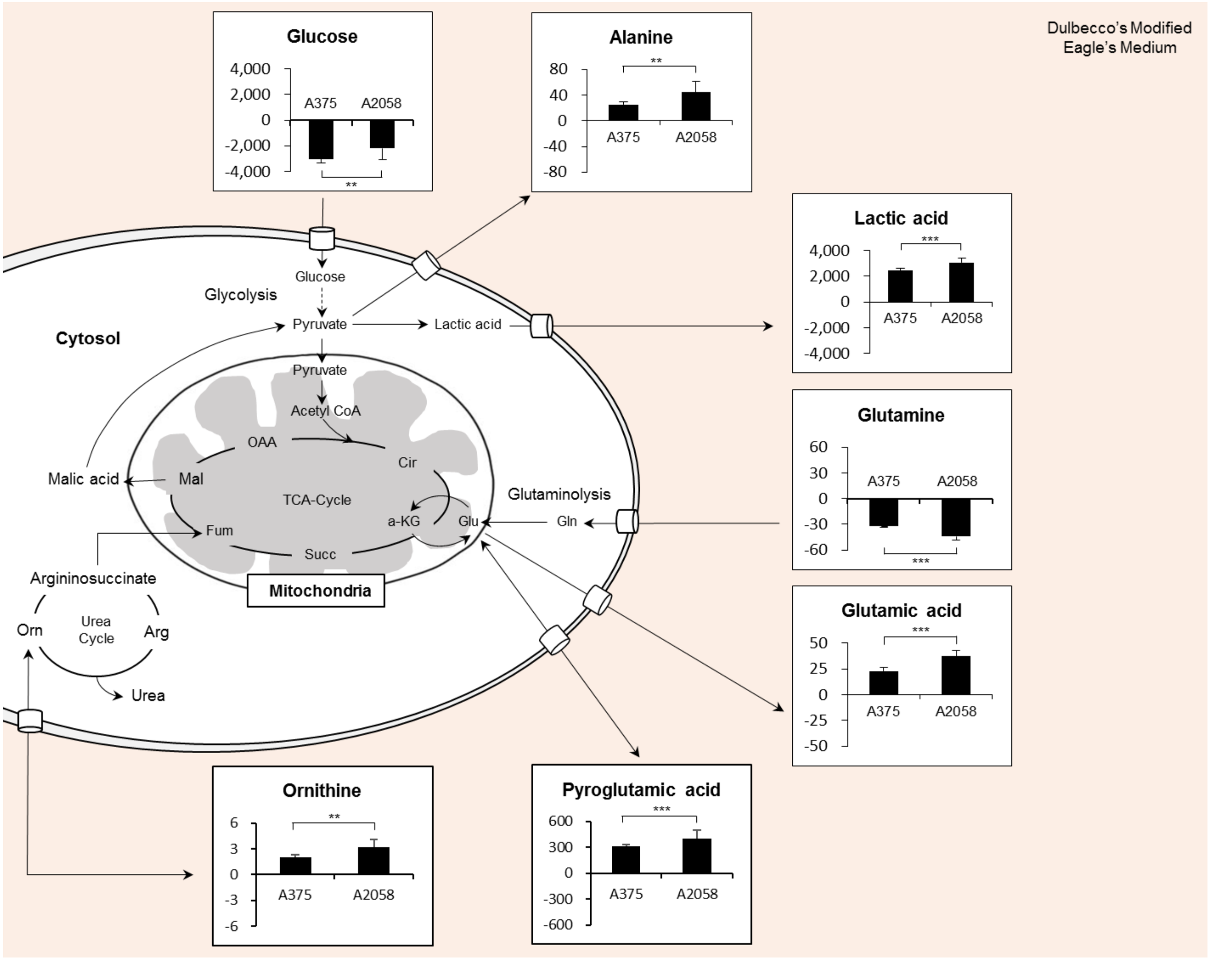

Supplementary Figure S3. Changes in the relative levels of glucose, alanine, lactic acid, glutamine, glutamic acid, ornithine, and pyroglutamic acid in fresh and spent media. Samples were taken fresh and spent media after 2 days of melanoma cell culture.
